# Different Oligomeric States of the Tumor Suppressor p53 Show Identical Binding Behavior towards the S100β Homodimer

**DOI:** 10.1002/cbic.202100665

**Published:** 2022-04-13

**Authors:** Alan An Jung Wei, Claudio Iacobucci, Wiebke Schultze, Christian H. Ihling, Christian Arlt, Andrea Sinz

**Affiliations:** ^1^ Department of Pharmaceutical Chemistry and Bioanalytics Center for Structural Mass Spectrometry Institute of Pharmacy Martin-Luther University Halle-Wittenberg Kurt-Mothes-Str. 3 06120 Halle/Saale Germany

**Keywords:** intrinsically disordered proteins, mass spectrometry, p53, S100β, tumor suppressors

## Abstract

The tumor suppressor protein p53 is a transcription factor that is referred to as the “guardian of the genome” and plays an important role in cancer development. p53 is active as a homotetramer; the S100β homodimer binds to the intrinsically disordered *C*‐terminus of p53 affecting its transcriptional activity. The p53/S100β complex is regarded as highly promising therapeutic target in cancer. It has been suggested that S100β exerts its oncogenic effects by altering the p53 oligomeric state. Our aim was to study the structures and oligomerization behavior of different p53/S100β complexes by ESI‐MS, XL‐MS, and SPR. Wild‐type p53 and single amino acid variants, representing different oligomeric states of p53 were individually investigated regarding their binding behavior towards S100β. The stoichiometry of the different p53/S100β complexes were determined by ESI‐MS showing that tetrameric, dimeric, and monomeric p53 variants all bind to an S100β dimer. In addition, XL‐MS revealed the topologies of the p53/S100β complexes to be independent of p53’s oligomeric state. With SPR, the thermodynamic parameters were determined for S100β binding to tetrameric, dimeric, or monomeric p53 variants. Our data prove that the S100β homodimer binds to different oligomeric states of p53 with similar binding affinities. This emphasizes the need for alternative explanations to describe the molecular mechanisms underlying p53/S100β interaction.

## Graphical abstract

**By combining** ESI‐MS, SPR, and XL‐MS, we investigated the interactions between the tumor suppressor p53 and S100β. Our approach unveiled binding of an S100β dimer to p53, independently from p53’s oligomeric state via similar molecular contacts and with comparable binding affinities. The molecular mechanism by which S100β regulates p53 activity goes beyond the currently proposed oligomerization inhibition mechanism.

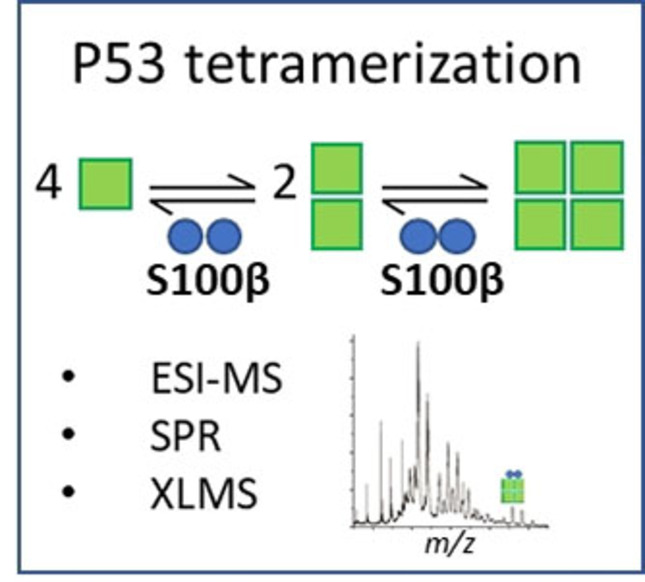

The tetrameric tumor suppressor protein p53 is a transcription factor that is induced by stress signals, such as DNA damage, oncogene activation, and nutrient deprivation. Apoptosis and cell‐cycle arrest are the two main outcomes of p53 activation.[^1^, ^2^] Protein binding partners of p53 play a crucial role as they not only contribute to p53 activation in response to cellular stress, but are also involved in p53 stabilization.^[3]^ A large number of protein binding partners interact with p53, predominantly via p53’s disordered *N*‐terminal transactivation domain and the disordered *C*‐terminal regulatory domain (REG), both of which are intrinsically disordered regions (IDRs).[^4^, ^5^] One prominent group of p53 binding partners is the S100 protein family^[6]^ that comprise a highly conserved group of EF‐hand motif‐containing, calcium‐binding proteins. Upon binding of calcium ions, a conformational change of S100 proteins is induced that enables binding to a variety of targets.^[7]^

A contradictory role of S100 binding to p53 has been described as S100 proteins display inhibitory effects on p53,^[8]^ but also showed a stimulatory effect on p53 in another study.^[9]^ One prominent member of the S100 protein family, S100β, is essential for p53‐mediated transcription as it exhibits a stabilizing effect upon binding to tetrameric p53.^[10]^ However, inhibition of p53’s transcriptional activity has also been reported after binding of S100β to monomeric p53 by a simultaneous prevention of p53 tetramer formation.^[11]^ Oligomerization of p53 is crucial as p53 binds to DNA in its functional tetrameric state. Each p53 tetramer presents a dimer of dimers where the primary dimer is formed via hydrogen bonds and salt bridges, and the tetramer is generated by hydrophobic interactions.^[12]^ Mutations in the tetramerization region (TET) can alter the oligomeric state of p53 and have so far been observed in various human cancers.^[13]^ By substituting a single amino acid, interactions between p53 monomers can be disrupted, thereby affecting oligomer formation. By introducing a single‐point mutation in p53’s TET (Figure 1a), alternative oligomeric states are induced in p53. This paves the way for conducting in‐depth binding studies between S100β and defined oligomeric states of p53.[^12^, ^14^]

**Figure 1:**
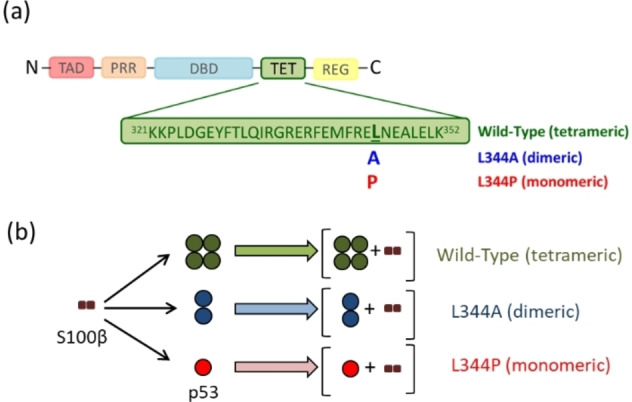
(a) Domain organization of full‐length p53. The amino acid sequence highlighting single amino acid exchanges in the tetramerization domain is presented. (b) Schematic view of the proposed stoichiometry between three p53 variants (tetrameric wild‐type, dimeric L344A variant, and monomeric L344P variant) and S100β.

Despite numerous studies dealing with the protein interactions between p53 and S100 proteins, the majority of these studies relies on the use of p53 peptides (Table S1),^[15]^ while investigations are still lacking for full‐length p53. In this work, we aim at elucidating the molecular details underlying the protein‐protein interactions between full‐length human p53 and S100β. For this, S100β (Figure S1) and three variants of full‐length p53 representing three different oligomeric states (tetrameric wild‐type, dimeric L344A variant, and monomeric L344P variant) were expressed in *E. coli* and purified (Figures S2–S4).^[16]^6ek; The stoichiometries and topologies of the p53/S100β complexes were investigated by ESI‐MS and XL‐MS. In addition, SPR studies were conducted to determine the binding affinities between p53 and S100β.

ESI‐MS provided specific insights into the stoichiometry of complexes formed between the three different p53 variants and S100β (Figure 1b). Due to the reported low binding affinity of p53/S100β in the micromolar range (Table S1),^[17]^ stabilizing the p53/S100β complexes is crucial to facilitate ESI‐MS experiments. Initial attempts to study the p53/S100β interaction via native MS were not successful as the complexes were not stable and acquiring mass spectra after buffer exchange was impossible (Figure S5). Therefore, mild cross‐linking between p53 and S100β was conducted to covalently fix both protein binding partners. To ensure that ESI‐MS experiments were carried out at native conditions we added the lowest possible concentration of cross‐linker disuccinimidyl dibutyric urea (DSBU), i. e., 0.4 equivalents of cross‐linker, compared to the number of nucleophilic residues (amine and hydroxy groups) in p53 and S100β (see Supporting Information). Apparently, our mild cross‐linking stabilization did not alter the charge state distribution of p53 tetramer (Figure 2a, 2b) as it matches the native p53 charge state distribution previously reported.^[18]^

**Figure 2:**
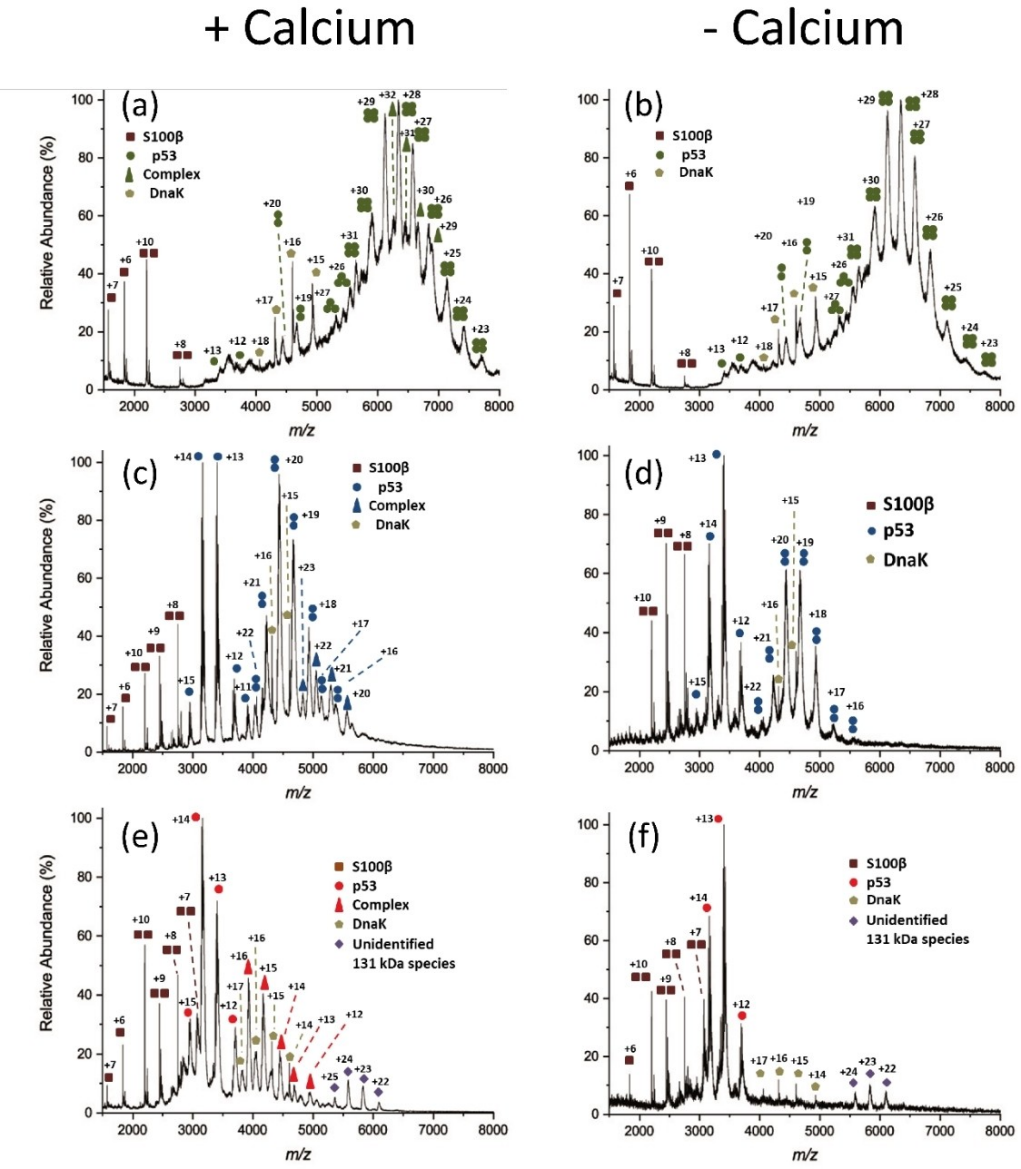
ESI mass spectra of DSBU‐modified full‐length p53/S100β complexes. The chaperon DnaK is always observed. (a) Wild‐type p53 in the presence of calcium; (b) Wild‐type p53 in the absence of calcium; (c) L344A p53 variant in the presence of calcium; (d) L344A p53 variant in the absence of calcium; (e) L344P p53 variant in the presence of calcium; (f) L344P variant in the absence of calcium. Zoom‐in presentations of mass spectra are shown in Figures S6–S8. Theoretical and experimental masses of different p53 and S100β species are shown in Table S2.

As the interaction between p53 and S100β is calcium‐dependent, one way to verify complex formation is to compare whether the p53/S100β complex is created in the presence or absence of calcium ions. ESI‐MS measurements of calcium‐loaded, DSBU‐modified wild‐type p53/S100β showed a charge state distribution ranging from +24 to +31 for tetrameric p53 and +29 to +32 for a 200‐kDa species (green triangle, Figure 2a). The identity of this high molecular weight species as a complex being composed of two S100β and four p53 units was further confirmed by collision‐induced dissociation (CID)^[19]^ tandem mass spectrometry (MS/MS) experiments.^[20]^ The ion at a mass‐to‐charge ratio (*m/z)* of 6661, indicating the presence of this complex, was subjected to collisional activation. Monomeric S100β subunits, monomeric p53 subunit, tetrameric p53 subunits, and peptide backbone fragments were ejected, confirming the stoichiometry of the complex (Figure 3a). In contrast, the mass spectrum of the sample containing wild‐type p53 and S100β in the absence of calcium (Figure 2b) displayed exclusively tetrameric wild‐type p53, but no signals of a p53/S100β complex.

**Figure 3:**
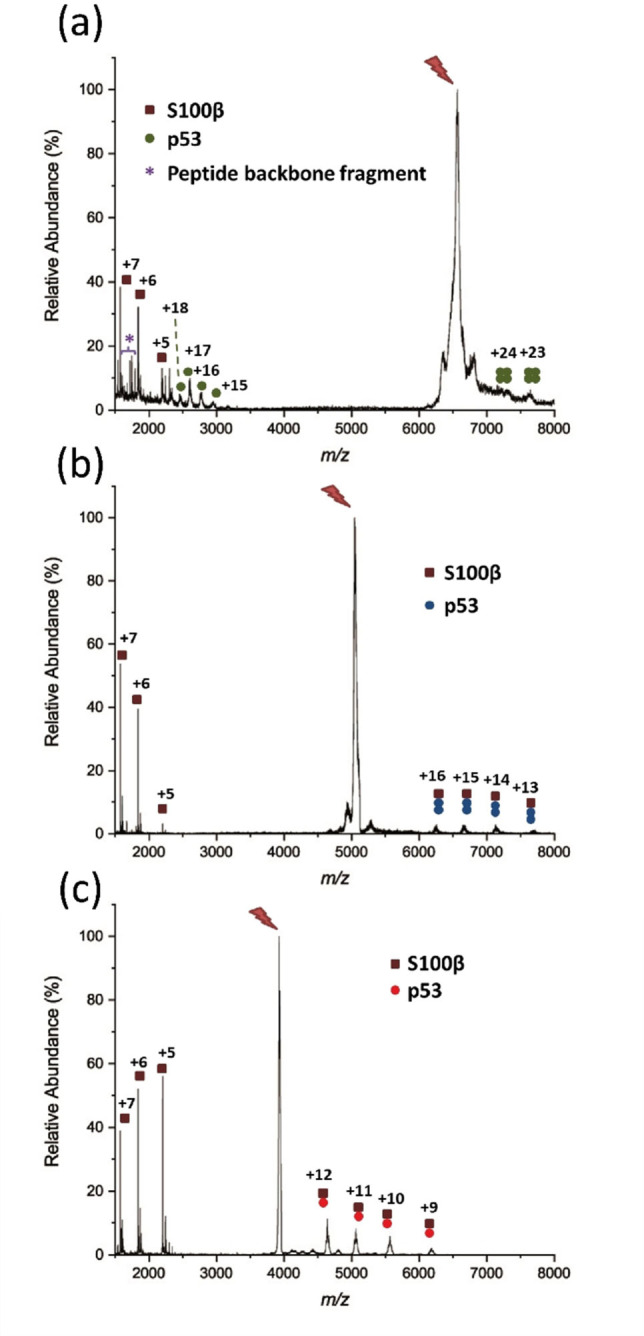
Tandem mass spectra (CID‐MS/MS) for p53/S100β complex. The precursor ion that was fragmented is marked with a red flash. (a) tetrameric wild‐type p53 (30+), signal at *m/z* 6661; (b) dimeric L344A p53 variant (22+), signal at *m/z* 5050; (c) monomeric L344P p53 variant (17+), signal at *m/z* 3927.

Comparable results were observed for the two p53 variants. For the dimeric p53 variant L344A, signals corresponding to the dimer were observed with a charge state distribution between +16 to +22. Also, a p53/S100β complex with a charge state distribution between +20 and +23 was observed, corresponding to a 111‐kDa species (blue triangle, Figure 2c). The assignment of these signals to a L344A p53/S100β (2 : 2) complex was confirmed by MS/MS experiments. The precursor ion at *m/z* 5050, representing the L344A p53/S100β (2 : 2) complex, was dissociated by CID‐MS/MS (Figure 3b). One monomeric S100β subunit was ejected from this dimeric p53/S100β complex leading to a remaining species comprising S100β monomer and p53 dimer. By comparing Figures 2c and 2d, it appears evident that the formation of the p53/S100β (2 : 2) complex did not significantly influence the oligomeric state of the L344A p53 variant as a similar charge state distribution pattern has also been observed for wild‐type tetrameric p53. Also, no p53/S100β complex formation was visible in the absence of calcium where only signals representing the p53 dimer were observed (Figure 2d). On the other hand, the monomeric L344P p53 variant/S100β complex showed a charge state distribution ranging between +14 to +17, corresponding to a species with a molecular weight of 66 kDa (red triangle, Figure 2e). In the absence of calcium, this complex was not observed (Figure 2f), which is identical to the behavior of the two other p53 variants. Tandem MS experiments again confirmed this species to correspond to a complex between an S100β dimer and a p53 monomer. The relevant precursor ion at *m/z* 3927 was subjected to CID‐MS/MS, yielding a 1 : 1 complex between p53 and S100β, together with an S100β monomer that was ejected upon collisional activation (Figure 3c). Signals corresponding to an unknown species were observed with a charge state distribution between +22 to +25 and with a mass range of *m/z* 5000–6500. The deconvoluted mass of this species corresponds to 131 kDa. The identity of this unknown species as non‐p53 related was confirmed by CID‐MS/MS experiments.

In contrast to previous reports where only interactions between tetrameric and monomeric p53 species were recorded,^[21]^ we observed an interaction of the dimeric p53 variant. Strikingly, p53/S100β complex formation does not appear to interfere with p53 oligomerization. This contradicts the currently existing hypothesis explaining the mechanism of p53 inhibition by S100β.^[21]^

The dissociation constants (K_D_ values) determined for selected p53 peptides from the tetramerization and regulatory domains of p53 (TET and REG) with S100β have so far been conducted with analytical ultracentrifugation (AUC) and fluorescence titration.[^15^, ^22^] In this study, we determined K_D_ values of the three full‐length p53/S100β complexes by SPR. For SPR measurements, S100β was immobilized on a carboxymethyldextran planar surface (CMDP). Each full‐length p53 variant was then injected individually at different concentrations and SPR sensograms were recorded (Figure 4). For tetrameric wild‐type p53, a dissociation constant of 41 μM (Figure 4a) was determined. For the dimeric p53 variant (L344A, Figure 4b), the K_D_ value was 60 μM, and for the monomeric p53 variant (L344P, Figure 4c) a dissociation constant of 40 μM was obtained. Despite a slightly lower affinity of S100β to the dimeric p53 variant L344A, compared to the other two p53 variants, the strength of the p53/S100β interaction appeared to be independent of the p53 oligomeric state. This is consistent with ESI‐MS findings (see above) indicating that the S100β dimer binds to tetrameric, dimeric, and monomeric states of p53.

**Figure 4:**
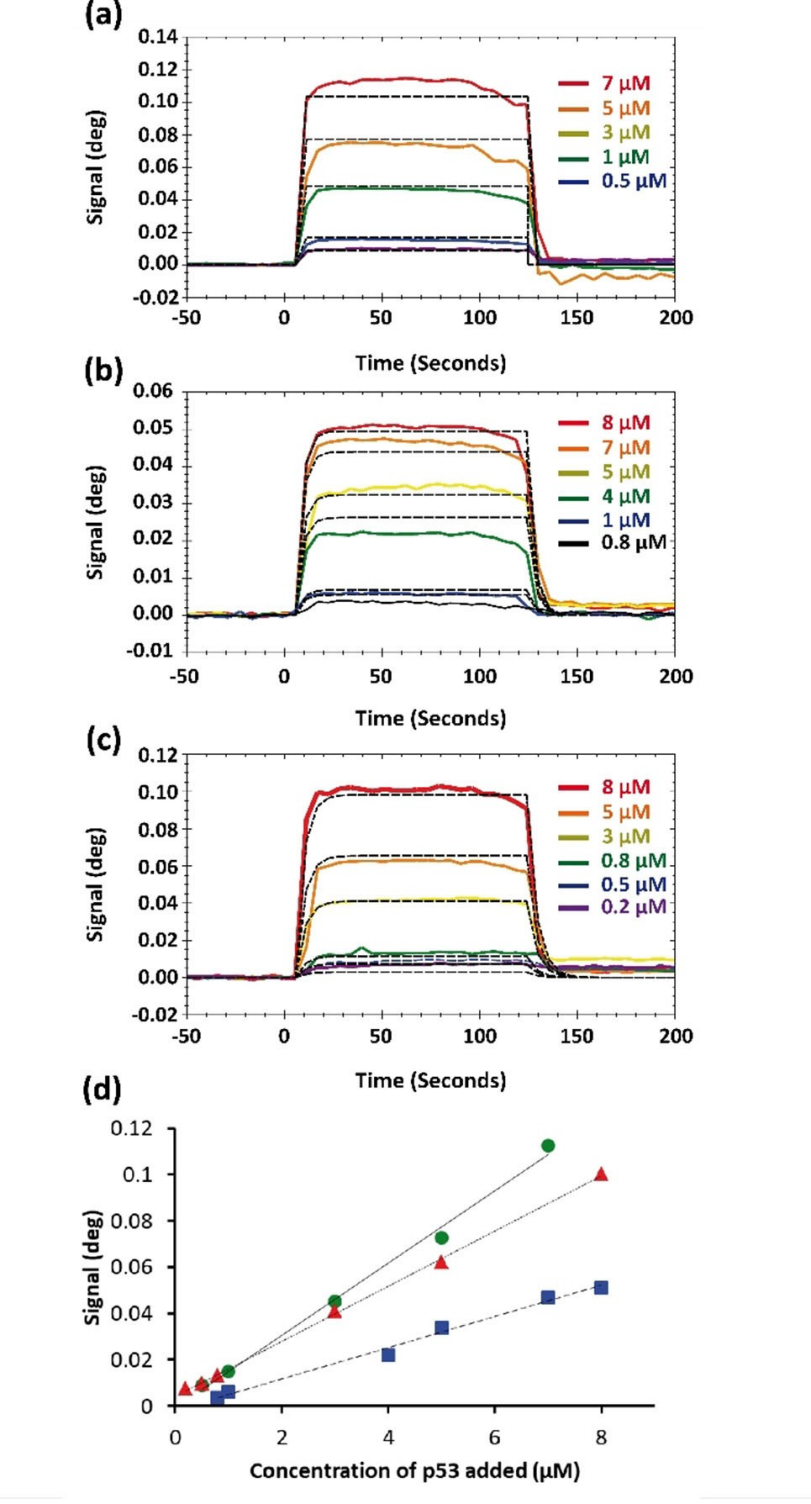
Surface plasmon resonance (SPR) experiments of p53 variants binding to immobilized S100β. (a) Wild‐type, tetrameric; (b) L344A dimeric; (c) L344P monomeric p53 with dashed lines showing curve fittings. The K_D_ value of wild‐type p53 and S100β is 41 μM. For dimeric p53 (L344A variant) the K_D_ value is 60 μM and for the monomeric p53 (L344P variant) the K_D_ value is 40 μM. (d) SPR experiments of three p53 variants binding to immobilized S100β. Wild‐type tetrameric p53 is plotted with green circles and a solid line, dimeric L344A p53 variant is plotted with blue squares and a dashed line, and monomeric L344P p53 variant is plotted with red triangles and a dotted line. For derived K_D_ values please see text.

After having determined the stoichiometries and binding affinities for the different p53/S100β complexes we sought to clarify the exact interaction sites between p53 and S100β by XL‐MS. Experiments were performed with three cross‐linkers possessing complementary reactivities and spacer lengths, namely the homo‐bifunctional amine‐reactive cross‐linker DSBU (12.5 Å), the amine‐carboxyl coupling reagent 1‐ethyl‐3‐(3‐dimethylaminopropyl)carbodiimide (EDC) / sulfo‐*N*‐hydroxysuccinimide (sulfo‐NHS) (0 Å), and the photo‐/amine‐reactive cross‐linker sulfosuccinimidyl 4,4′‐azipentanoate (sulfo‐SDA) (3.9 Å). Using these three complementary reagents allowed targeting all p53 and S100β regions. To better visualize the site‐specific interaction, mapping of cross‐links was illustrated with xiNET (Figure 5).^[23]^ We first verified by one‐dimensional gel electrophoresis (SDS‐PAGE) that our cross‐linking conditions (see Supporting Information for experimental details) did indeed capture the correct oligomeric states of p53/S100β complexes for all p53 variants (Figures S9–S11). The three different cross‐linking principles invariantly connected two S100β monomers to either the wild‐type tetrameric, L344A dimeric or L344P monomeric p53, confirming the stoichiometry determined by ESI‐MS. Subsequent in‐depth LC/MS/MS analyses of the p53/S100β complexes delivered a total of eight unique inter‐protein cross‐linking sites between wild‐type p53 and S100β, 7 unique cross‐linking sites between L344A p53 variant and S100β, and 8 unique cross‐linking sites between L344P p53 variant and S100β (Figures S12–S14). Selected MS/MS spectra allowed an unambiguous identification of cross‐linked amino acids (Figures S15–S17). All unique cross‐linking sites identified between p53 and S100β for the different cross‐linkers are summarized in Supporting Information Tables S3, S4, S5, and S6 and mapping of cross‐links is displayed in Figure 5.

**Figure 5:**
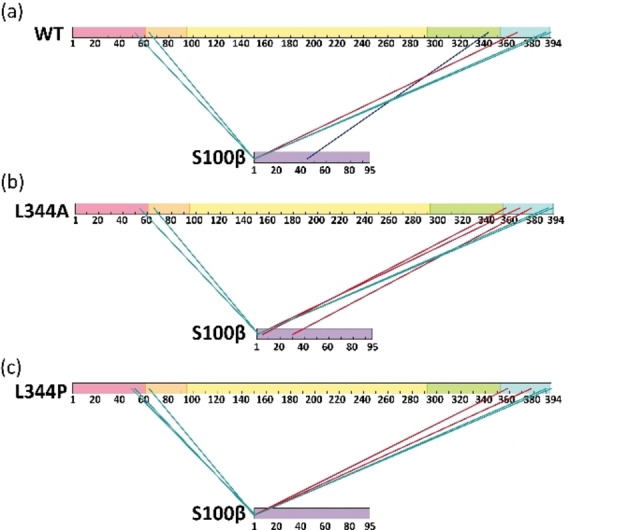
Mapping of cross‐links between p53 and S100β (a) tetrameric wild‐type p53, (b) dimeric L344A p53 variant, and (c) monomeric L344P p53 variant. Red lines indicate DSBU, cyan lines indicate EDC/NHS, and blue lines indicate sulfo‐SDA. Cross‐link sites are shown in Tables S3–S5.

The vast majority of interprotein cross‐links between p53 and S100β connect the *N*‐terminus of S100β with either the TAD or REG domains of p53, regardless of the oligomeric state of p53 (Figure 5). It has to be noted that the cross‐linking sites found for S100β involve an artificial *N*‐terminus of three amino acids (Gly‐Ser‐His) that remained on S100β after thrombin cleavage (see Supporting Information). We ruled out that these three additional amino acids at the *N*‐terminus of S100β might interfere with the overall topology of p53/S100β complex by repeating the XL‐MS experiments with recombinant, tag‐free human S100β protein. Identical cross‐links with p53 were identified for both S100β preparations (Figures S18–S20; Table S6). Strikingly, the cross‐links identified were highly similar, suggesting a similar topology of all three p53/S100β complexes, independently of the oligomeric state of the respective p53 variants and confirms ESI‐MS and SPR data.

Our integrated experimental approach, combining ESI‐MS, SPR, and XL‐MS, provides a solid basis for an in‐depth characterization of the molecular interactions between p53 and S100β. ESI‐MS revealed that two S100β monomers bind to wild‐type p53 tetramer, L344A p53 dimer, and L344P p53 monomer, via similar molecular contacts. Most importantly, the interaction with S100β was found to be independent of the oligomeric state of p53. S100β was shown to exhibit a comparable binding affinity towards all p53 oligomeric states under investigation. Conclusively, it is tempting to speculate that the molecular mechanism, by which S100β regulates the activity of p53, is apparently not determined by p53’s oligomeric state, which contrasts currently existing knowledge. Further experiments will have to prove how the interaction of p53 and S100β is regulated.

## Experimental Section

Experimental procedures are provided in detail in the Supporting Information. MS data have been deposited to the ProteomeXchange Consortium via the PRIDE partner repository with the project accession PXD029914, username: reviewer_pxd030001@ebi.ac.uk; password: 0JIKK9rp.

Expression and purification of p53 and S100β were performed according to previously described methods.[^16^, ^18^, ^24^] ESI‐MS was conducted after mild cross‐linking of p53 and S100β. Buffer exchange of the DSBU‐modified samples of two variants (L344P and L344A) to 500 mM ammonium acetate (pH 6.8) was performed with Amicon Ultra centrifugal filter units (MWCO 30 kDa, Merck Millipore). For wild‐type p53, an online buffer exchange (OBE) system with Ultimate 3000 RSLC nano‐HLPC system (Thermo Fisher Scientific; Figure S21). ESI‐MS experiments were performed with a High‐Mass Q‐TOF II instrument (Micromass/MS Vision). XL‐MS experiments were performed with DSBU, sulfo‐SDA, and EDC/NHS incubated at 4 °C. Samples were then analyzed with timsTOF Pro mass spectrometer. SPR experiments were performed with the MP‐SPR Navi 200 OTSO instrument (BioNavis) (Figure S22). Immobilization of S100β was performed on a CMDP sensor slide (Xantac) and injections of three p53 variants (L344P, L344A, and wild‐type) were performed individually.

## Conflict of interest

The authors declare no conflict of interest.

## Supporting information

As a service to our authors and readers, this journal provides supporting information supplied by the authors. Such materials are peer reviewed and may be re‐organized for online delivery, but are not copy‐edited or typeset. Technical support issues arising from supporting information (other than missing files) should be addressed to the authors.

Supporting Information

## Data Availability

MS data have been deposited to the ProteomeXchange Consortium via the PRIDE partner repository with the project accession PXD029914, username: reviewer_pxd030001@ebi.ac.uk; password: 0JIKK9rp.

## References

[1] J. Chen , Cold Spring Harbor Perspect. Med. 2016, 6, a026104. 10.1101/cshperspect.a026104 PMC4772082 26931810

[2] E. Yonish-Rouach , C. Choisy , V. Deguin , C. Breugnot , E. May , Behring Inst. Mitt. 1996, 97, 60–71. 8950467

[3] M. F. Lavin , N. Gueven , Cell Death Differ. 2006, 13, 941–950. 16601750 10.1038/sj.cdd.4401925

[4] B. Xue , C. J. Brown , A. K. Dunker , V. N. Uversky , Biochim. Biophys. Acta Proteins Proteomics 2013, 1834, 725–738. 10.1016/j.bbapap.2013.01.012 PMC3905691 23352836

[5] A. C. Joerger , A. R. Fersht , Cold Spring Harbor Perspect. Biol. 2010, 2, a000919. 10.1101/cshperspect.a000919 PMC2869527 20516128

[6] J. Van Dieck , T. Brandt , D. P. Teufel , D. B. Veprintsev , A. C. Joerger , A. R. Fersht , Oncogene 2010, 29, 2024–2035. 20140014 10.1038/onc.2009.490

[7] M. R. Fernandez-Fernandez , T. J. Rutherford , A. R. Fersht , Protein Sci. 2008, 17, 1663–1670. 18694925 10.1110/ps.035527.108 PMC2548378

[8] J. Lin , M. Blake , C. Tang , D. Zimmer , R. R. Rustandi , D. J. Weber , F. Carrier , J. Biol. Chem. 2001, 276, 35037–35041. 11454863 10.1074/jbc.M104379200

[9] C. Scotto , C. Delphin , J. C. Deloulme , J. Baudier , Mol. Cell. Biol. 1999, 19, 7168–7180. 10490652 10.1128/mcb.19.10.7168 PMC84710

[10] M. V. Blagosklonny , Oncogene 1997, 15, 1889–1893. 9365234 10.1038/sj.onc.1201374

[11] S. E. Kern , J. A. Pietenpol , S. Thiagalingam , A. Seymour , K. W. Kinzler , B. Vogelstein , Science 1992, 256, 827–830. 1589764 10.1126/science.1589764

[12] J. Gencel-Augusto , G. Lozano , Genes Dev. 2020, 34, 1128–1146. 32873579 10.1101/gad.340976.120 PMC7462067

[13] Y. Itahana , H. Ke , Y. Zhang , J. Biol. Chem. 2009, 284, 5158–5164. 19106109 10.1074/jbc.M805696200 PMC2643511

[14] J. van Dieck , M. R. Fernandez-Fernandez , D. B. Veprintsev , A. R. Fersht , J. Biol. Chem. 2009, 284, 13804–13811. 19297317 10.1074/jbc.M901351200 PMC2679481

[15] M. R. Fernandez-Fernandez , D. B. Veprintsev , A. R. Fersht , Proc. Natl. Acad. Sci. USA 2005, 102, 4735–4740. 15781852 10.1073/pnas.0501459102 PMC555715

[16] C. Arlt , C. H. Ihling , A. Sinz , Proteomics 2015, 15, 2746–2755. 25728495 10.1002/pmic.201400549

[17] R. R. Rustandi , A. C. Drohat , D. M. Baldisseri , P. T. Wilder , D. J. Weber , Biochemistry 1998, 37, 1951–1960. 9485322 10.1021/bi972701n

[18] C. Arlt , V. Flegler , C. H. Ihling , M. Schäfer , I. Thondorf , A. Sinz , Angew. Chem. Int. Ed. 2017, 56, 275–279; 10.1002/anie.201609826 27897373

[19] J. L. P. Benesch , J. Am. Soc. Mass Spectrom. 2009, 20, 341–348. 19110440 10.1016/j.jasms.2008.11.014

[20] K. Pagel , S. J. Hyung , B. T. Ruotolo , C. V. Robinson , Anal. Chem. 2010, 82, 5363–5372. 20481443 10.1021/ac101121r

[21] J. van Dieck , M. R. Fernandez-Fernandez , D. B. Veprintsev , A. R. Fersht , J. Biol. Chem. 2009, 284, 13804–13811. 19297317 10.1074/jbc.M901351200 PMC2679481

[22] C. Delphin , M. Ronjat , J. C. Deloulme , G. Garin , L. Debussche , Y. Higashimoto , K. Sakaguchi , J. Baudier , J. Biol. Chem. 1999, 274, 10539–10544. 10187847 10.1074/jbc.274.15.10539

[23] C. W. Combe , L. Fischer , J. Rappsilber , Mol. Cell. Proteomics 2015, 14, 1137–1147. 25648531 10.1074/mcp.O114.042259 PMC4390258

[24] M. H. Clare, Bachelor Thesis, *Rekombinante Expression von S100β und Interaktionsstudien mit dem Tumorsupressor p53*, Martin-Luther Universität Halle-Wittenberg (Germany), **2018**.

