# Supplementary material for: Different Oligomeric States of the Tumor Suppressor p53 Show Identical Binding Behavior towards the S100β Homodimer
Source: Chembiochem. 2022 Apr 13;23(11):e202100665. doi: 10.1002/cbic.202100665 (PMC9400850; doi:10.1002/cbic.202100665)
Supplement: Supplementary file 1 — Supporting Information [file CBIC-23-0-s001.pdf]

ChemBioChem

Supporting Information

Different Oligomeric States of the Tumor Suppressor p53 Show Identical Binding Behavior towards the S100β Homodimer

Alan An Jung Wei, Claudio Iacobucci,* Wiebke Schultze, Christian H. Ihling, Christian Arlt,* and Andrea Sinz*

Supporting Information

Different Oligomeric States of the Tumor Suppressor p53 Show Identical Binding Behavior Towards the S100β Homodimer

Alan An Jung Wei[a], Claudio Iacobucci[a*], Wiebke Schultze[a], Christian H. Ihling[a], Christian Arlt[a*], and Andrea Sinz[a*]

a A. Wei, W. Schultze, Dr. C. Iacobucci, Dr. C. Arlt, Prof. Dr. A. Sinz
Department of Pharmaceutical Chemistry and Bioanalytics, Center for Structural Mass Spectrometry,
Institute of Pharmacy, Martin-Luther University Halle-Wittenberg
Kurt-Mothes-Str. 3, D-06120 Halle/Saale, Germany

[*] E-mail: andrea.sinz@pharmazie.uni-halle.de; christian.arlt@pharmazie.uni-halle.de; iacobucci.claudio@gmail.com

Table of Content

Page Number

Materials and Methods ……………………………………………………………………………………………………3

- Protein Expression and Purification
- Covalent Modification of Proteins
- Buffer Exchange
- ESI-MS
- Cross-linking of Proteins
- Enzymatic Digestion
- Nano-HPLC-MS/MS
- MS Data Analysis
- Surface Plasmon Resonance (SPR)

Amino acid Sequences ……………………………………………………………………………………………..7

Additional Figures and Tables ……………………………………………………………………………….….9

Literature References ………………………………………………………………………………………………39

Materials and Methods

Protein Expression and Purification

Expression and purification of full-length human p53 (wild-type, L344A, L344P) were performed as previously described[1].

The sequence of full-length human S100β was synthesized as codon-optimized gene for recombinant expression in *Escherichia coli* by GeneArt. For cloning, the full-length human S100β sequence with *N*-terminal $(His)_6$ tag and thrombin cleavage site were incorporated into the pET28(+) plasmid with cleavage sites *Xho*I and *Nde*I. *Escherichia coli* BL21(DE3) cells were transformed with the ligated plasmid[2]. Cells were induced at OD_{600}=1.2 with 1 mM isopropyl β-d-1-thiogalactopyranoside (IPTG) and harvested after overnight incubation at 37°C. Protein was purified (ÄKTA pure, GE Healthcare) by immobilized metal-ion affinity chromatography (IMAC) (1 mL HisTrap FF column, GE Healthcare) in 50 mM 2-[4-(2-hydroxyethyl)-piperazine-1-yl]ethanesulfonic acid (HEPES), 300 mM NaCl, 2.5 mM Tris(2-carboxyethyl)phosphine hydrochloride (TCEP), and 0-500 mM imidazole at pH=8.0 followed by size exclusion chromatography (SEC) (HiLoad™ 16/600 Superdex™ 200pg, Cytiva) in 50 mM 2-[4-(2-hydroxyethyl)-piperazine-1-yl]ethanesulfonic acid (HEPES), 300 mM NaCl, 2.5 mM Tris(2-carboxyethyl)phosphine hydrochloride (TCEP), and 10% v/v glycerol at pH=7.2. Recombinant, tag-free human S100β protein was obtained from ProSpec (PRO-2312).

All chemicals were obtained from Sigma at the highest purity if not stated otherwise. Milli-Q-water was used for all reactions.

Covalent Modification of Proteins

N-hydroxysuccinimide (NHS) esters, such as disuccinimidyl dibutyric urea (DSBU, CF Plus Chemicals), mainly react with the primary amines of lysines and the *N*-termini of proteins. Reactions with amino acids that contain hydroxy groups, such as serines, threonines, and tyrosines, are also observed. A recent comparative community-wide cross-linking mass spectrometry (XL-MS) study[3] reported that serines, threonines, and tyrosines account for ~30% of NHS ester reaction sites. In human p53, here are 21 primary amine groups and 69 hydroxy groups there are 90 potential reactive sites of p53. The same calculation for S100β with 8 primary amine groups and 9 hydroxy groups results in 17 potential reactive sites. Conclusively, the molar concentration of nucleophilic residues in solution is [p53] x $p53_{\text{reactive sites}}$ + [S100β] x $S100\beta_{\text{reactive sites}}$ = 6 µM x 42 + 6 µM x 11 = 318 µM. Thus, the molar ratio of DSBU over the nucleophilic residues in solution is 120 µM / 318 µM = 0.38, corresponding to ~ 0.4 equivalents of the cross-linker DSBU.

All covalent modification experiments were performed at 4°C in buffer (50 mM HEPES, 300 mM NaCl, 2.5 mM TCEP, and 10% glycerol, pH 7.2). Each variant of p53 (wild-type, L344A, and L344P) was used at a final concentration of 6 µM, S100β was used at a final concentration of 6 µM, and DSBU with a final concentration of 0.12 mM (20-fold molar excess of p53). 1 mM of calcium chloride was added to calcium-containing DSBU-modified samples, while for calcium-depleted DSBU-modified samples 1 mM of the chelating agent EGTA was added. Quenching of cross-linking reactions was performed by adding 20 mM of ammonium bicarbonate after 60 minutes. Buffer exchange was conducted immediately after the addition of ammonium bicarbonate.

Buffer Exchange

Prior to ESI-MS experiments, buffer exchange to 500 mM ammonium acetate (pH 6.8) was performed. Buffer exchange of the cross-linked samples (L344P and L344A variants of p53) was performed with Amicon Ultra centrifugal filter units (molecular weight cut-off 30 kDa, Merck Millipore). For the cross-linked samples of wild-type p53, an online buffer exchange (OBE) procedure was performed. The sample was loaded via the autosampler on the Ultimate 3000 RSLC nano-HLPC system (Thermo Fischer Scientific). The sample was then desalted and buffer exchanged with a self-packed polyacrylamide P6 column (BioRad).

ESI-MS

All ESI-MS experiments were performed with a High-Mass Q-TOF II instrument (Micromass/MS Vision). Experiments were measured with following instrumental settings: Capillary voltage, 2 – 2.2 kV (for online buffer-exchanged samples), 1.2 – 1.4 kV (for manually buffer-exchanged samples with Amicon filters); sample cone voltage, 60-80 V; collision energy, 30-100 V; source pressure, 1.0 x 10^{-1} mbar; collision cell pressure, 1.0 x 10^{-2} mbar – 1.2 x 10^{-2} mbar. The transmission range was set between *m/z* 1000-8000. For collision-induced dissociation tandem mass spectrometry (CID-MS/MS), the collision energy was set to 120 – 140 V.

Cross-linking of Proteins

All cross-linking reactions were performed in three replicates and analyzed separately.

DSBU cross-linking

A solution containing 6 µM wild-type p53 or L344A or L344P variants of p53 and 6 µM S100β in buffer (50 mM HEPES, 1 mM $CaCl_2$, 300 mM NaCl, and 2.5 mM TCEP, pH 7.2) was cross-linked at 4°C for 1 h with DSBU at 100-fold molar excess. DSBU was dissolved in neat dimethyl sulfoxide (DMSO) at a concentration of 0.6 mM, immediately before adding it to the protein solution. The reaction was quenched by adding ammonium bicarbonate to a final concentration of 20 mM.

EDC/sulfo-NHS cross-linking

A solution containing 6 µM wild-type p53 or L344A or L344P variants of p53 and 6 µM S100β in buffer (50 mM HEPES, 1 mM $CaCl_2$, 300 mM NaCl, and 2.5 mM TCEP, pH 7.2) was cross-linked at 4°C for 2 h with 1-ethyl-3-(3-dimethylaminopropyl)carbodiimide (EDC) and sulfo-*N*-hydroxysuccinimide (sulfo-NHS) (both Thermo Fisher) at 500-fold molar excess. EDC was dissolved in neat DMSO at a concentration of 3 mM, immediately before adding it to the protein solution. Sulfo-NHS was dissolved in a buffer containing 50 mM HEPES, 1 mM $CaCl_2$, 300 mM NaCl, and 2.5 mM TCEP, pH 7.2) at a concentration of 3 mM. The reaction was quenched by adding ammonium bicarbonate to a final concentration of 20 mM.

Sulfo-SDA crosslinking

A solution containing 6 µM wild-type p53 or L344A or L344P variants of p53 and 6 µM S100β in buffer (50 mM HEPES, 1 mM $CaCl_2$, 300 mM NaCl, and 2.5 mM TCEP, pH 7.2) was cross-linked at 4°C for 2 hrs with EDC and sulfo-NHS at 500-fold molar excess. Sulfosuccinimidyl 4,4'-azipentanoate

(sulfo-SDA) was dissolved in DMSO at a concentration of 0.6 mM, immediately before adding it to the protein solution. After the reaction with EDS/sulfo-NHS the sample was irradiated for 15 s at 365 nm (Panasonic Aicure UJ30/35 in a home-built irradiation chamber. The reaction was quenched by adding ammonium bicarbonate to a final concentration of 20 mM.

Enzymatic Digestion

All cross-linked samples were reduced to a volume of 2-3 µl in a SpeedVac concentrator. 25 µl of 8 M urea in 400 mM ammonium bicarbonate was added to each sample. After 5 min of sonication, samples were incubated with dithiothreitol (DTT, 6.4 mM) for 30 min at 56°C and iodoacetamide (12.5 mM) for 20 min at room temperature in the dark. The alkylation reaction was quenched by adding 9 mM of DTT. After diluting the samples to 1 M urea with water, they were incubated overnight at 37°C with AspN (NEB) at an 1:20 (w/w) enzyme-substrate ratio and digested with trypsin (Promega) at 1:20 (w/w) for 4 h at 37 °C. All proteolytic digestions were stopped by adding 10% (v/v) TFA.

Nano-HPLC-MS/MS

Digested samples were analyzed by nano-HPLC-MS/MS on an UltiMate 3000 RSLC nano-HPLC system (Thermo Fisher Scientific) coupled to a timsTOF Pro mass spectrometer equipped with CaptiveSpray source (Bruker Daltonik). Peptides were trapped on a C18 column (precolumn Acclaim PepMap 100, 300 µm × 5 mm, 5 µm, 100 Å) (Thermo Fisher Scientific) and separated on a µPAC 50-cm column (PharmaFluidics). After trapping, peptides were eluted by a linear 90-min gradient from 3% (v/v) to 40% (v/v) ACN. For elution, a flow gradient was employed ranging from 900 to 600 nl/min in 15 min, followed by a constant flow rate of 600 nl/min. The column was washed at a flow rate of 600 nl/min with the following gradient: 40% (v/v) to 85% (v/v) ACN (5 min), 85% (v/v) ACN (5 min), 85% (v/v) to 3% (v/v) ACN (5 min). All separations were performed at room temperature.

For the settings of the timsTOF Pro mass spectrometer, the following parameters were adapted, starting with the parallel accumulation serial fragmentation (PASEF) method for standard proteomics. The values for mobility-dependent collision energy ramping were set to 95 eV at an inversed reduced mobility ($1/k_0$) of 1.6 V s/cm^2 and 23 eV at 0.73 V s/cm^2. Collision energies were linearly interpolated between these two $1/k_0$ values and kept constant above or below. No merging of TIMS scans was performed. Target intensity per individual PASEF precursor was set to 20 000. The scan range was set between 0.6 and 1.6 V s/cm^2 with a ramp time of 166 ms. 14 PASEF MS/MS scans were triggered per cycle (2.57 s) with a maximum of seven precursors per mobilogram. Precursor ions in an m/z range between 100 and 1700 with charge states ≥2+ and ≤8+ were selected for fragmentation. Active exclusion was enabled for 0.4 min (mass width 0.015 Th, 1/k0 width 0.015 V s/cm^2).

Data Analysis

Identification of cross-links was performed with MeroX 2.0.1.7. The following settings were applied: Proteolytic cleavage: *C*-terminal at Lys and Arg for (3 missed cleavages were allowed) and *N*-terminal at Asp and Glu (3 missed cleavages were allowed); peptide lengths of 5 to 30 amino acids; modifications: alkylation of Cys by iodoacetamide (fixed), oxidation of Met (variable); cross-linker specificity: Lys, Ser, Thr, Tyr, *N*-terminus (DSBU), Lys, N-terminus, Asp, Glu, *C*-terminus (EDC/sulfo-NHS); Lys, *N*-terminus, Asp, Glu, C-terminus (sulfo-SDA); search algorithm: RISEUP mode with up to three missing ions mode with minimum peptide score of 0; precursor mass accuracy: 10 ppm;

fragment ion mass accuracy: 20 ppm; signal-to-noise ratio > 1.5; precursor mass correction enabled; 10% intensity as prescore cut-off; 1% false discovery rate (FDR) cut-off, and minimum score cut-off: 50. Mass recalibration was performed for all files.

MS data have been deposited to the ProteomeXchange Consortium via the PRIDE partner repository with the project accession PXD029914, username: reviewer_pxd030001@ebi.ac.uk; password: 0JIKK9rp.

Mapping of cross-links was visualized with xiNET[4].

Surface Plasmon Resonance (SPR)

All SPR measurements were acquired with MP-SPR Navi 200 OTSO instrument (BioNavis). A CMDP sensorchip (Xantac) was used to perform experiments to investigate the protein-protein binding interactions between p53 (tetrameric wild-type, dimeric variant L344A, monomeric variant L344P) and S100β. All buffers were degassed and filtered prior to SPR experiments. Pre-concentration experiments were performed in order to test the optimal immobilization pH (3.6, 3.9, 4.2) of 10 mM sodium acetate, where a pH value of 3.9 was found to be the most optimal.

The running buffer for ligand immobilization and binding experiments consisted of 50 mM HEPES, 300 mM NaCl, 2.5 mM TCEP, and 1 mM of $CaCl_2$ (pH 7.2). For protein immobilization, 5 µM of S100β was diluted with 10 mM sodium acetate buffer (pH 3.9). The buffer for immobilizing S100β was 10 mM sodium acetate (pH 3.9). The amine coupling chemistry was applied for immobilization by using EDC/NHS to activate the CMDP surface and 1 M ethanolamine to deactivate non-reacted NHS-esters on the sensor surface.

Full-length p53 (wild-type, L344A, L344P) was injected separately at the following concentrations: 0.5, 1, 3, 5, 7 µM (wild-type p53), 0.8, 1, 4, 5, 7, 8 µM (L344A), 0.2, 0.5, 0.8, 3, 5, 8 µM (L344P). The running buffer consisted of 50 mM HEPES, 300 mM NaCl, 2.5 mM TCEP, and 1 mM calcium chloride. Prior to injection of each concentration, a regeneration buffer with 10 mM NaOH and 10 mM EGTA was used to remove bound analyte. Data processing was performed with the software TraceDrawer (BioNavis) where the on/off association (K_a)/dissociation (K_d) constants were used to calculate the dissociation constant (K_D).

Amino Acid Sequences

Amino acid sequence of human p53

Wild-Type (tetrameric p53 variant)

MEEPQSDPSV EPPLSQETFS DLWKLLPENN VLSPLPSQAM DDLMLSPDDI

EQWFTEDPGP DEAPRMPEAA PPVAPAPAAP TPAAPAPAPS WPLSSSVPSQ

KTYQGSYGFR LGFLHSGTAK SVTCTYSPAL NKMFCQLAKT CPVQLWVDST

PPPGTRVRAM AIYKQSQHMT EVVRRCPHHE RCSDSDGLAP PQHLIRVEGN

LRVEYLDDRN TFRHSVVVPY EPPEVGSDCT TIHYNYMCNS SCMGGMNRRP

ILTIITLEDS SGNLLGRNSF EVRVCACPGR DRRTEEENLR KKGEPHHELP

PGSTKRALPN NTSSSPQPKK KPLDGEYFTL QIRGRERFEM FRE**L**NEALEL

KDAQAGKEPG GSRAHSSHLK SKKGQSTSRH KKLMFKTEGP DSD

L344A (dimeric p53 variant)

MEEPQSDPSV EPPLSQETFS DLWKLLPENN VLSPLPSQAM DDLMLSPDDI

EQWFTEDPGP DEAPRMPEAA PPVAPAPAAP TPAAPAPAPS WPLSSSVPSQ

KTYQGSYGFR LGFLHSGTAK SVTCTYSPAL NKMFCQLAKT CPVQLWVDST

PPPGTRVRAM AIYKQSQHMT EVVRRCPHHE RCSDSDGLAP PQHLIRVEGN

LRVEYLDDRN TFRHSVVVPY EPPEVGSDCT TIHYNYMCNS SCMGGMNRRP

ILTIITLEDS SGNLLGRNSF EVRVCACPGR DRRTEEENLR KKGEPHHELP

PGSTKRALPN NTSSSPQPKK KPLDGEYFTL QIRGRERFEM FRE**A**NEALEL

KDAQAGKEPG GSRAHSSHLK SKKGQSTSRH KKLMFKTEGP DSD

L344P (monomeric p53 variant)

MEEPQSDPSV EPPLSQETFS DLWKLLPENN VLSPLPSQAM DDLMLSPDDI

EQWFTEDPGP DEAPRMPEAA PPVAPAPAAP TPAAPAPAPS WPLSSSVPSQ

KTYQGSYGFR LGFLHSGTAK SVTCTYSPAL NKMFCQLAKT CPVQLWVDST

PPPGTRVRAM AIYKQSQHMT EVVRRCPHHE RCSDSDGLAP PQHLIRVEGN

LRVEYLDDRN TFRHSVVVPY EPPEVGSDCT TIHYNYMCNS SCMGGMNRRP

ILTIITLEDS SGNLLGRNSF EVRVCACPGR DRRTEEENLR KKGEPHHELP

PGSTKRALPN NTSSSPQPKK KPLDGEYFTL QIRGRERFEM FREPNEALEL

KDAQAGKEPG GSRAHSSHLK SKKGQSTSRH KKLMFKTEGP DSD

Amino acid sequence of human S100β

The *N*-terminal GSH tag is highlighted in red.

GSHMSELEKA MVALIDVFHQ YSGREGDKHK LKKSELKELI NNELSHFLEE

IKEQEVVDKV METLDNDGDG ECDFQEFMAF VAMVTTACHE FFEHE

Additional Figures and Tables

SDS-PAGE of S100β purification

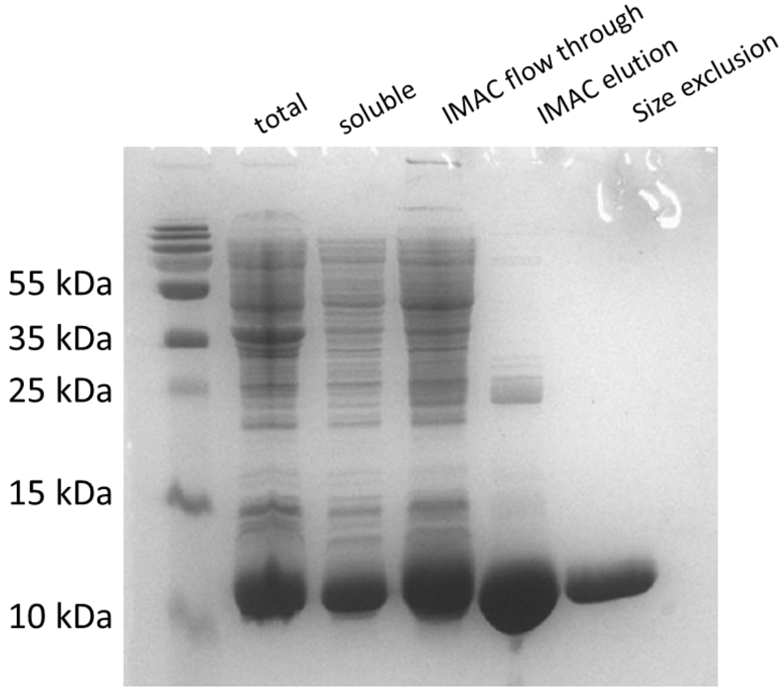

Figure S1. SDS-PAGE of the S100β protein purification

SDS-PAGE of p53 purification

<u>Wild-Type</u>

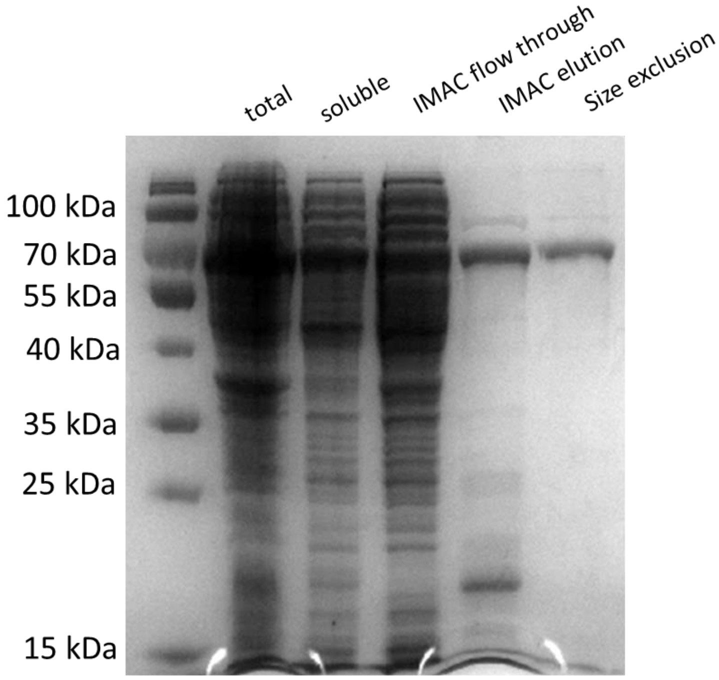

Figure S2. SDS-PAGE of the p53 (Wild-Type, tetrameric) protein purification

L344A

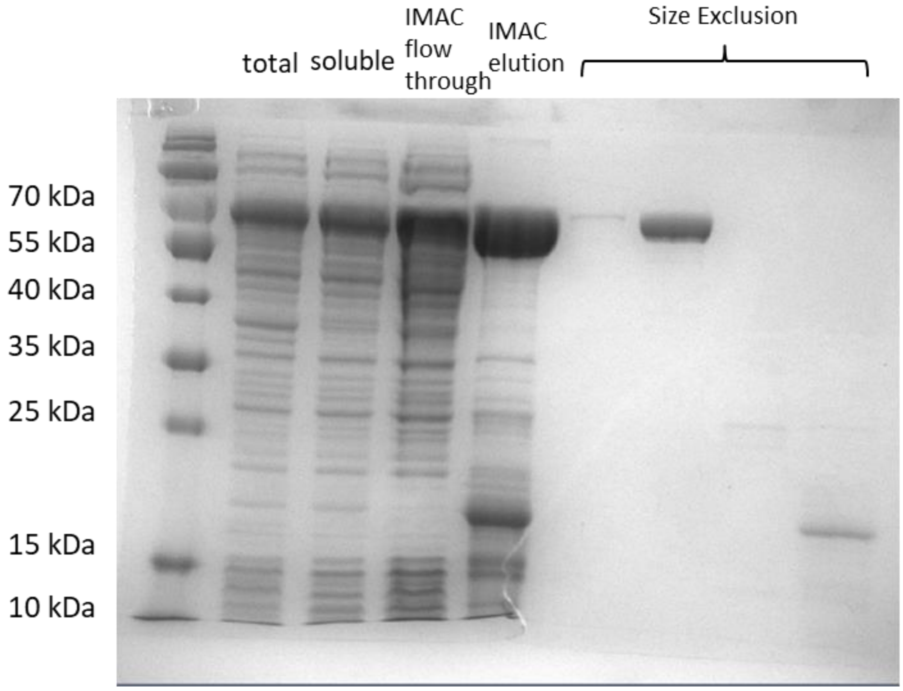

Figure S3. SDS-PAGE of the p53 (L344A, dimeric variant) protein purification

L344P

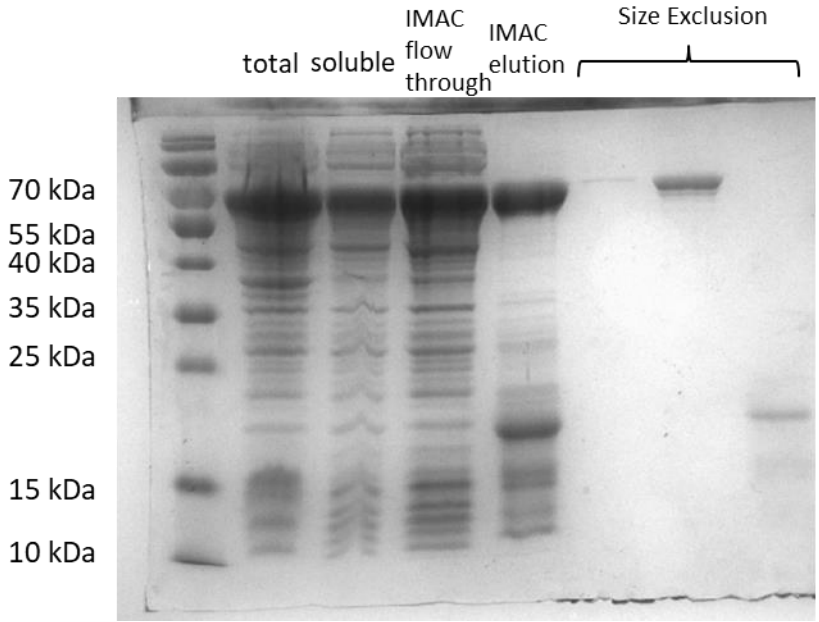

Figure S4. SDS-PAGE of the p53 (L344P, monomeric variant) protein purification

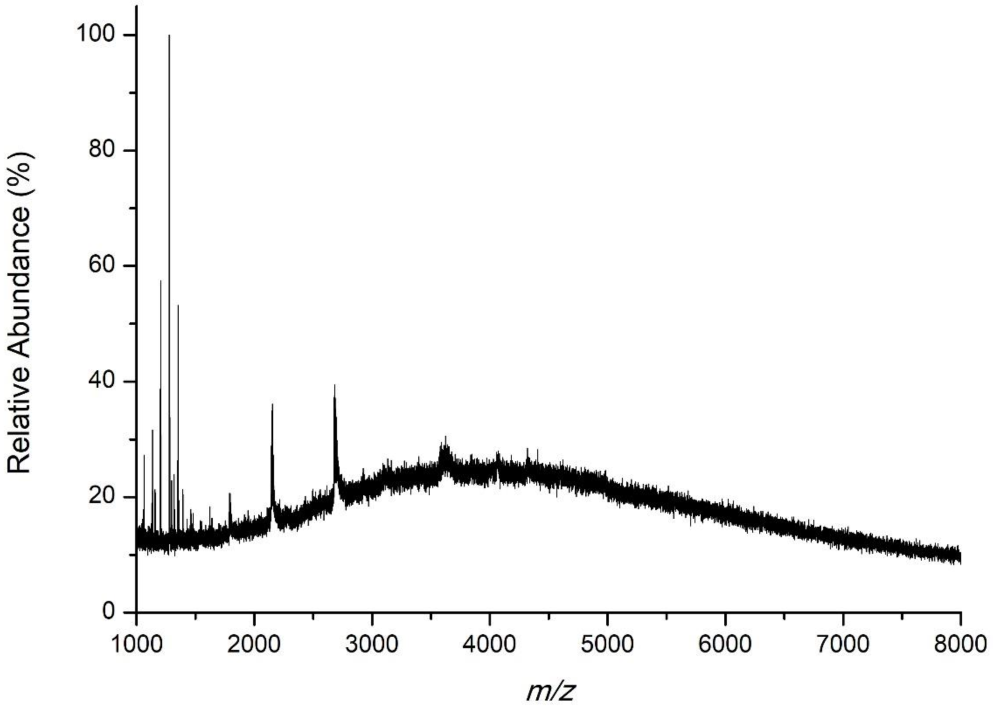

Figure S5. ESI-MS of wild-type p53 and S100β without DSBU modification.

Wild-Type (tetrameric)

	m/z	
+32	6260.024	(200288 Da)
+31	6461.591	(200278 Da)
+30	6659.522	(199755 Da)
+29	6881.975	(199548 Da)
	~200 kDa	

(One p53 tetramer + one S100β dimer)

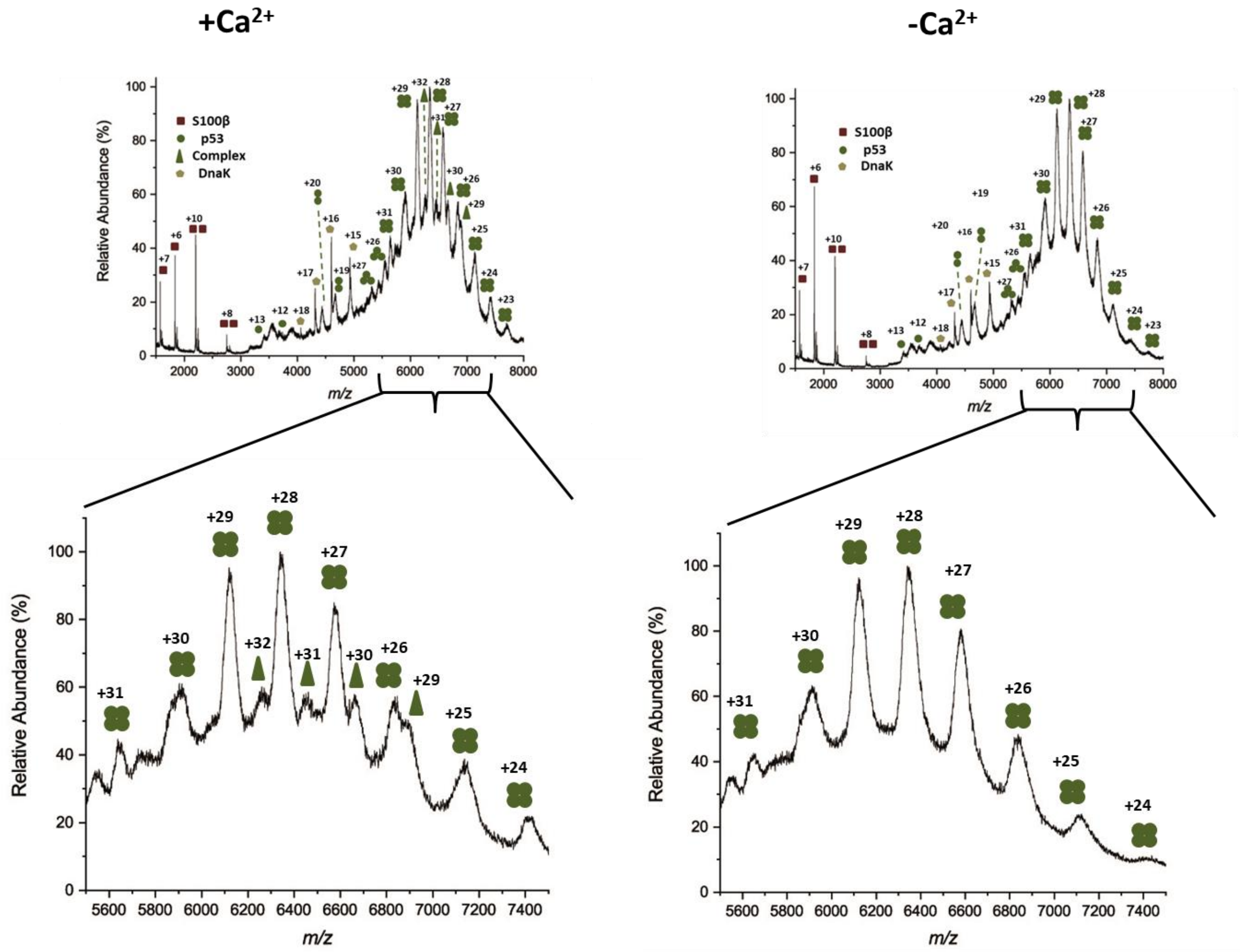

Figure S6. ESI-MS spectra show complex formation of S100β and wild-type tetrameric p53. Peak assignments of calcium-added and -depleted samples are presented.

L344A (dimeric)

		m/z	
+23	▲	4829.25	(111049 Da)
+22	▲	5049.26	(111061 Da)
+21	▲	5289.60	(111061 Da)
+20	▲	5562.60	(111232 Da)
	▲		(111 kDa)

(one p53 dimer + one S100B dimer)

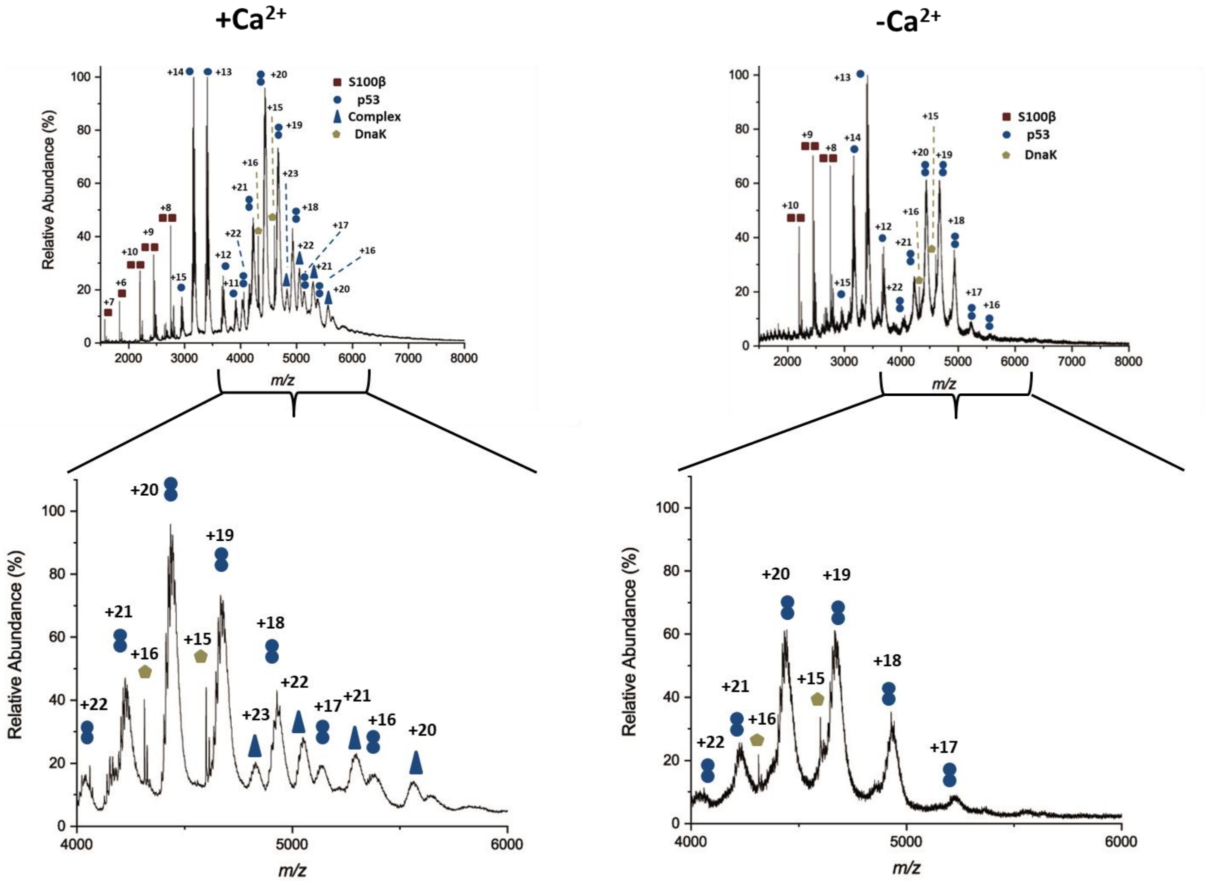

Figure S7. ESI-MS spectra show complex formation of S100β and dimeric p53 L344A variant. Peak assignments of calcium-added and -depleted samples are presented.

L344P (monomeric)

		m/z	
+16	▲	3922.58	(66666 Da)
+15	▲	4169.30	(66692 Da)
+14	▲	4446.41	(66681 Da)
+13	▲	4693.71	(65697 Da)
	▲		66 kDa

(One p53 monomer + one S100β dimer)

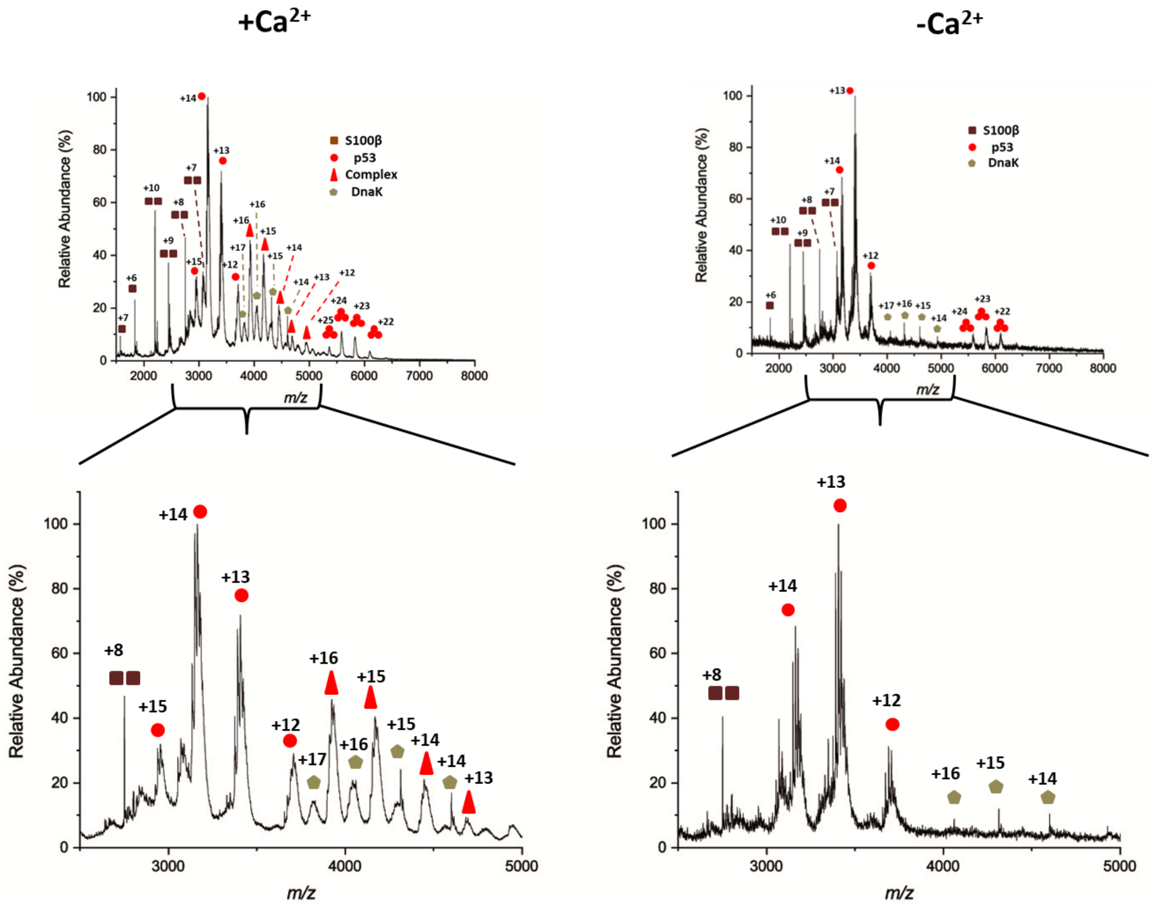

Figure S8. ESI-MS spectra show complex formation of S100β and monomeric p53 L344P variant. Peak assignments of calcium-added and -depleted samples are presented.

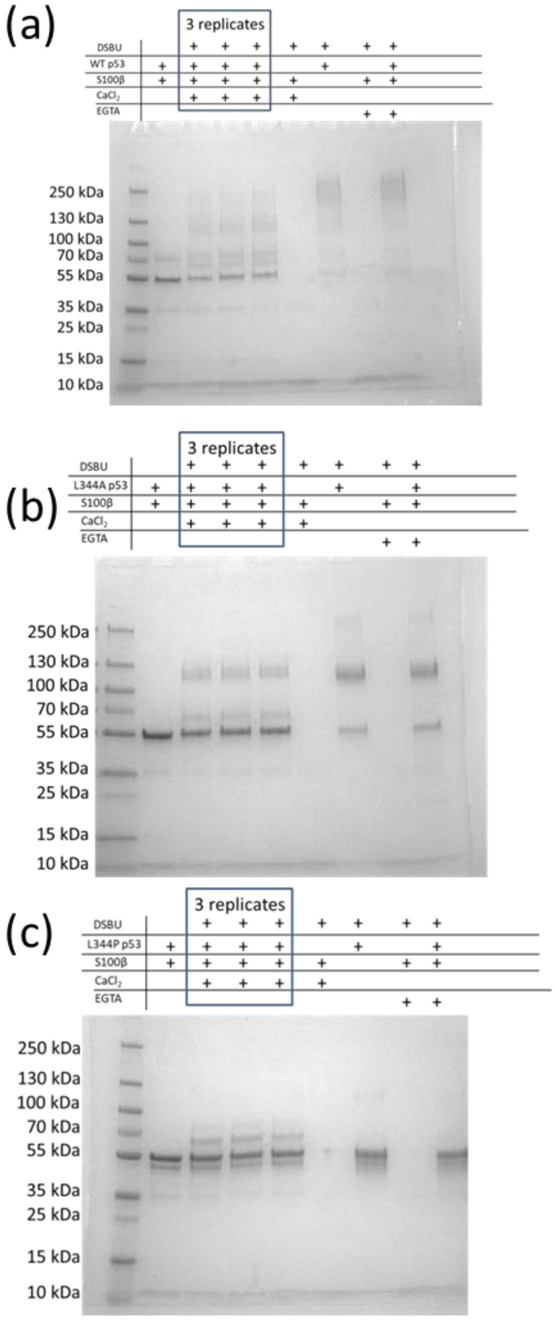

Figure S9. SDS-PAGE (4-20% gradient gel) of XL experiments of S100β and p53 with DSBU; (a) tetrameric wild-type, (b) dimeric L344A, and (c) monomeric L344P p53.

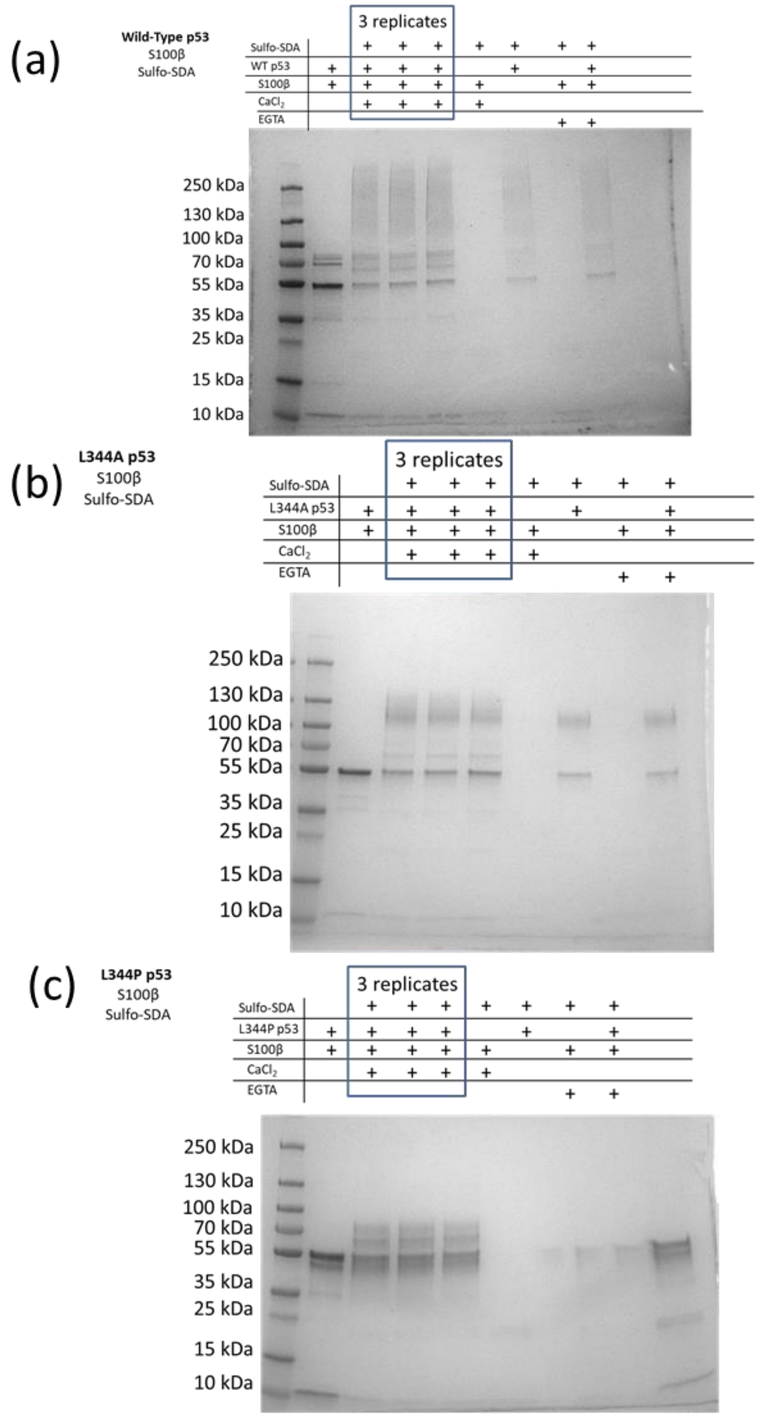

Figure S10. SDS-PAGE (4-20% gradient gel) for XL experiments of S100β and p53 with sulfo-SDA; (a) tetrameric wild-type, (b) dimeric L344A, and (c) monomeric L344P p53.

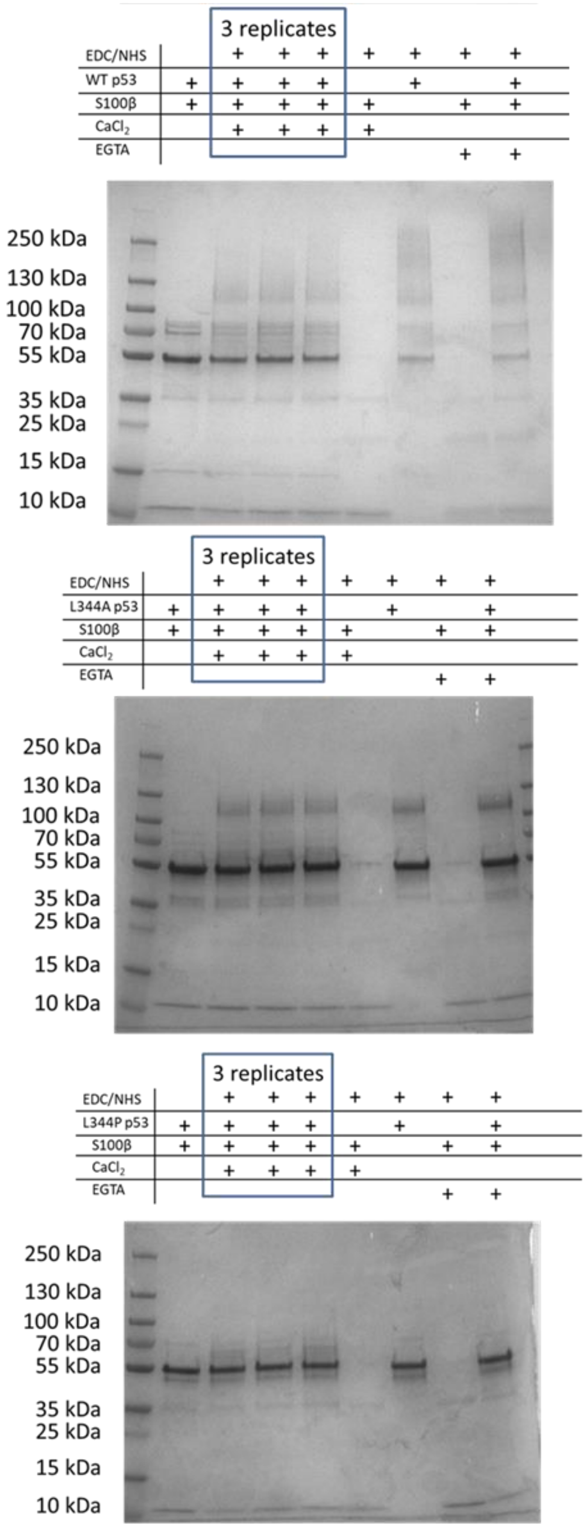

Figure S11. SDS-PAGE (4-20% gradient gel) for cross-linking experiments of S100β and p53 with EDC/NHS; (a) tetrameric wild-type, (b) dimeric L344A, and (c) monomeric L344P p53.

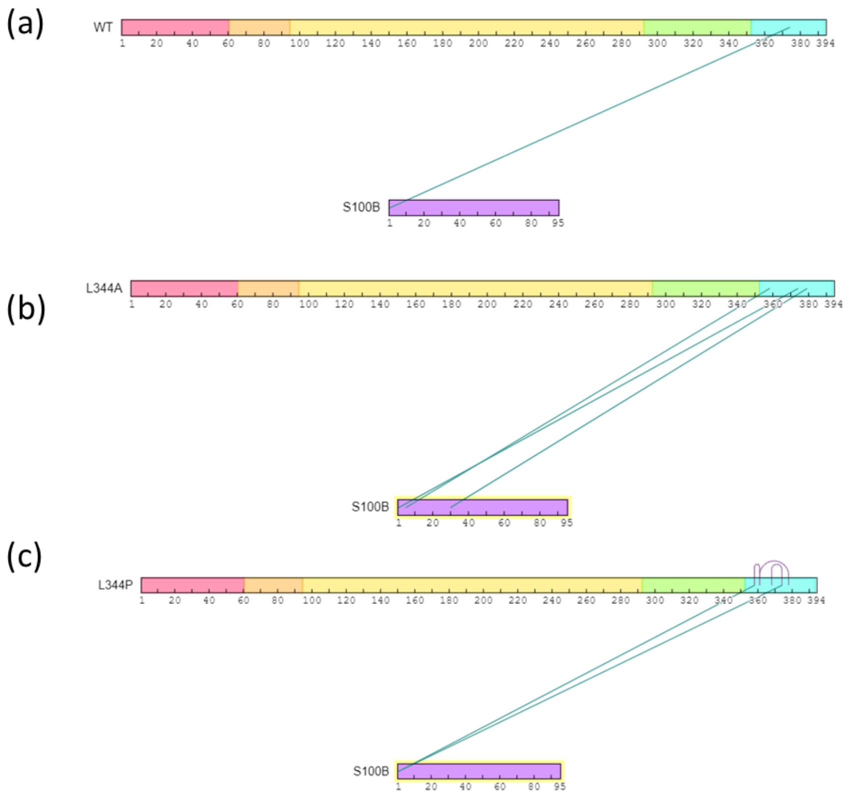

Figure S12. Mapping of DSBU cross-linked samples of S100β and (a) tetrameric wild-type, (b) dimeric L344A, and (c) monomeric L344P p53. Cross-links are visualized with xiNET[4].

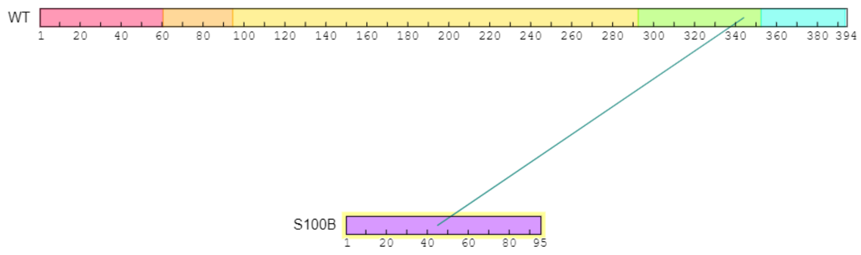

Figure S13. Mapping of sulfo-SDA cross-linked samples of S100β and tetrameric wild-type p53. Cross-links are visualized with xiNET[4].

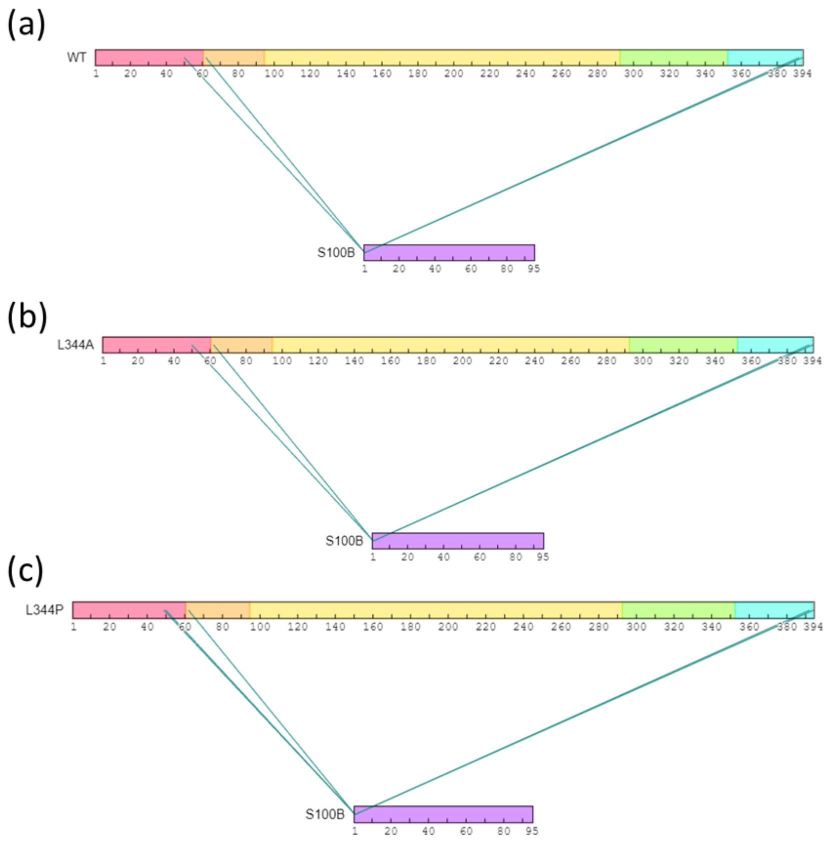

Figure S14. Mapping of EDC/NHS cross-linked samples of S100β and (a) tetrameric wild-type, (b) dimeric L344A, and (c) monomeric L344P p53. Cross-links are visualized with xiNET[4].

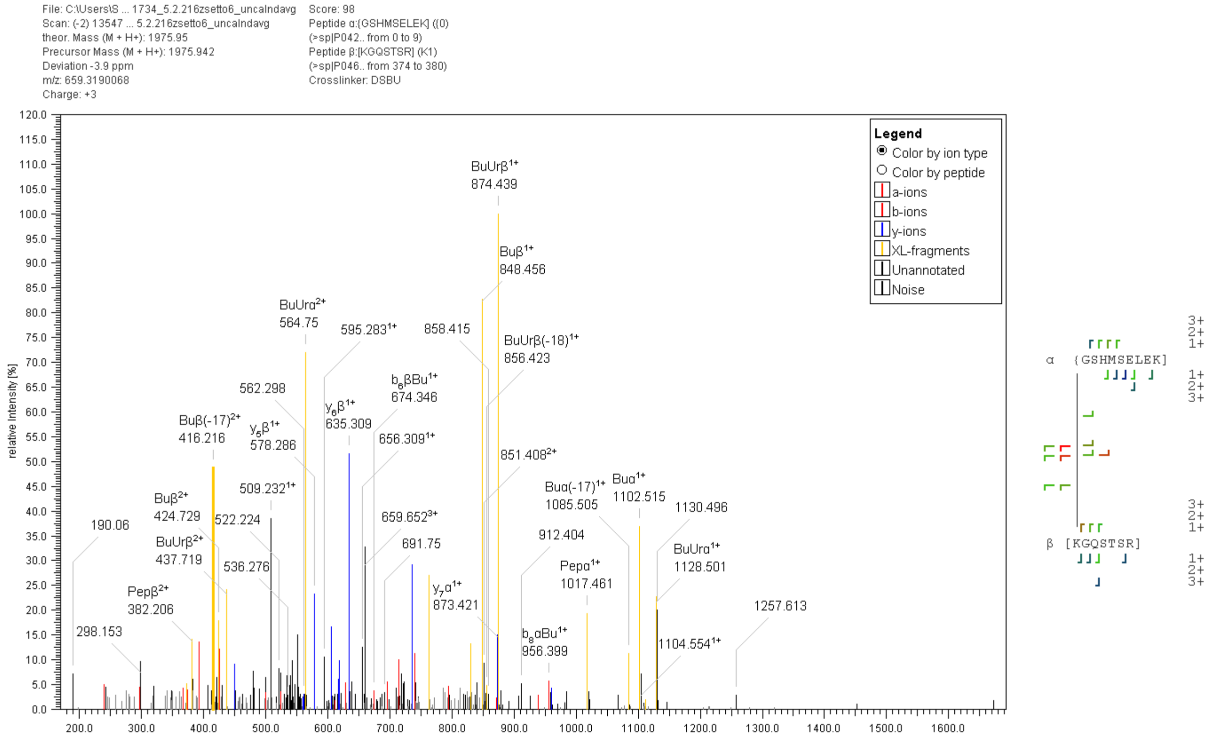
File: C:\Users\S ... 1734_5.2.216zsetto6_uncalndavg
Scan: (-2) 13547 ... 5.2.216zsetto6_uncalndavg
theor. Mass (M + H+): 1975.95
Precursor Mass (M + H+): 1975.942
Deviation -3.9 ppm
m/z: 659.3190068
Charge: +3
Score: 98
Peptide α:[GSHMSELEK] ([0)
(>sp|P042.. from 0 to 9)
Peptide β:[KGQSTSR] (K1)
(>sp|P046.. from 374 to 380)
Crosslinker: DSBU
Legend
Color by ion type
Color by peptide
a-ions
b-ions
y-ions
XL-fragments
Unannotated
Noise
relative Intensity [%]
α [GSHMSELEK]
β [KGQSTSR]

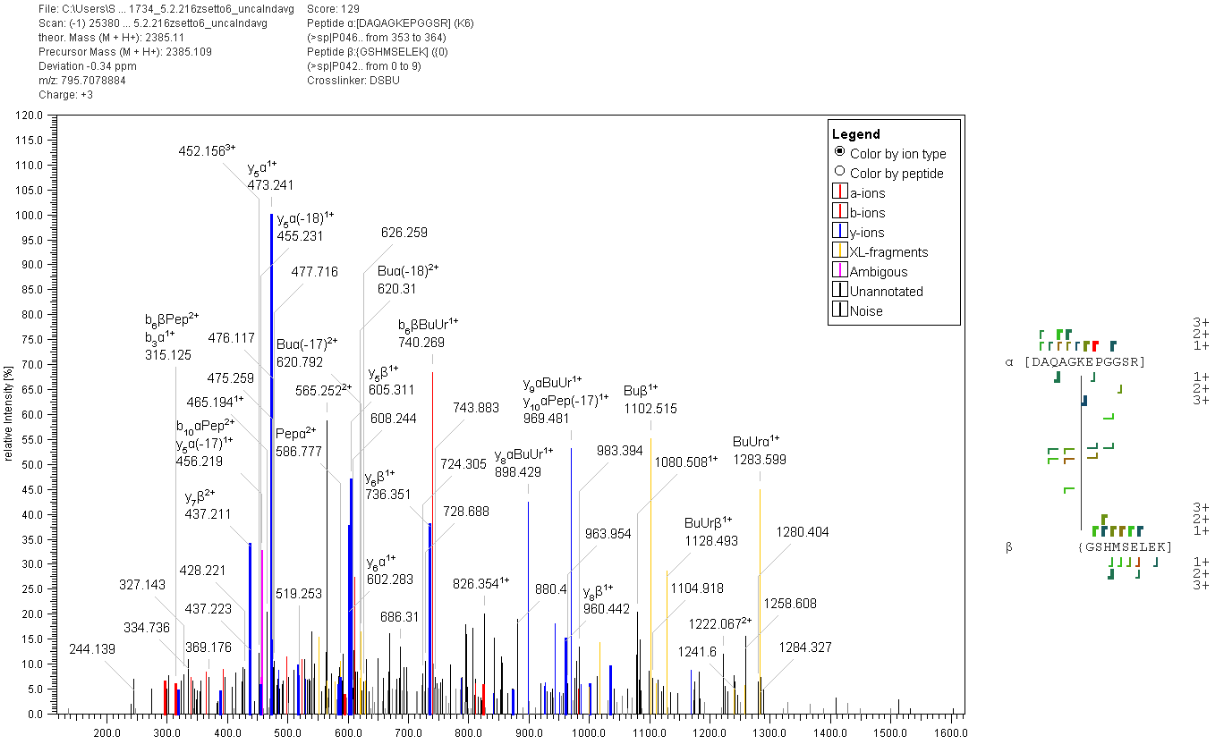
File: C:\Users\S ... 1734_5.2.216zsetto6_uncalndavg
Scan: (-1) 25380 ... 5.2.216zsetto6_uncalndavg
theor. Mass (M + H+): 2385.11
Precursor Mass (M + H+): 2385.109
Deviation -0.34 ppm
m/z: 795.7078884
Charge: +3
Score: 129
Peptide α:[DAQAGKEPGGSR] (K6)
(>sp|P046.. from 353 to 364)
Peptide β:[GSHMSELEK] ([0)
(>sp|P042.. from 0 to 9)
Crosslinker: DSBU
Legend
Color by ion type
Color by peptide
a-ions
b-ions
y-ions
XL-fragments
Ambigous
Unannotated
Noise
relative Intensity [%]
α [DAQAGKEPGGSR]
β [GSHMSELEK]

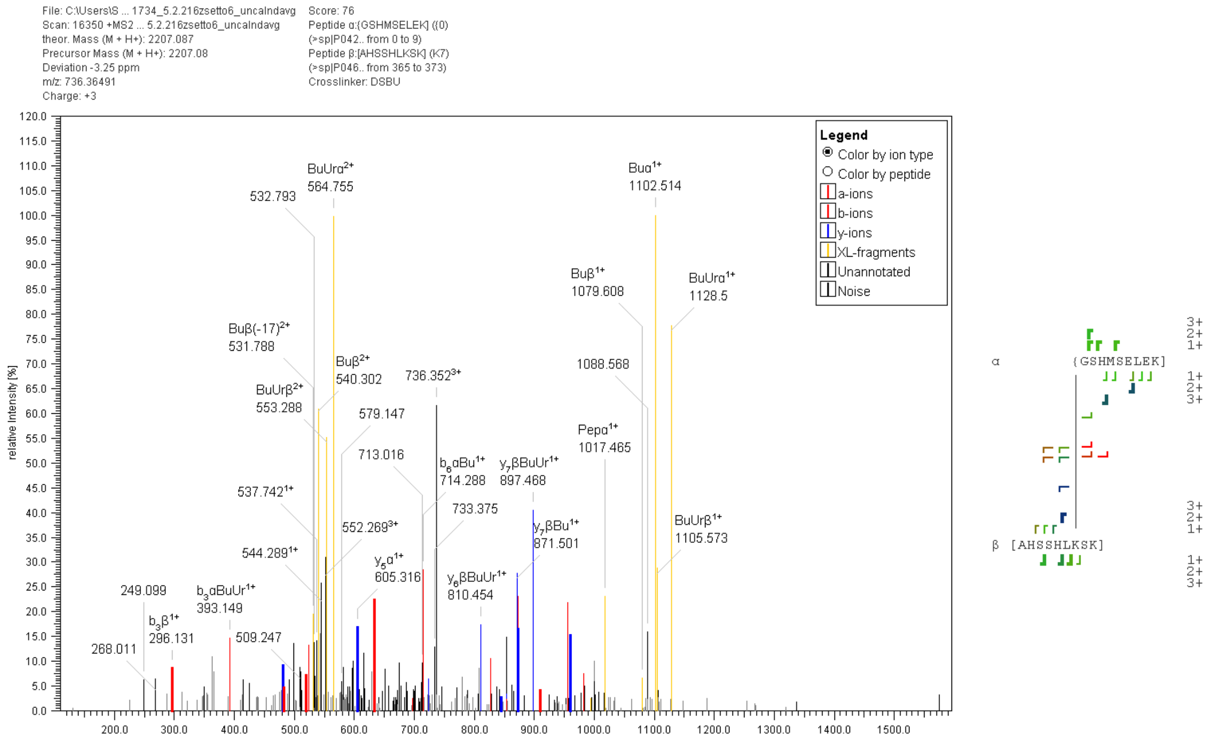

Figure S15. Selected MS/MS spectra and cross-linking assignment of DSBU with MeroX.

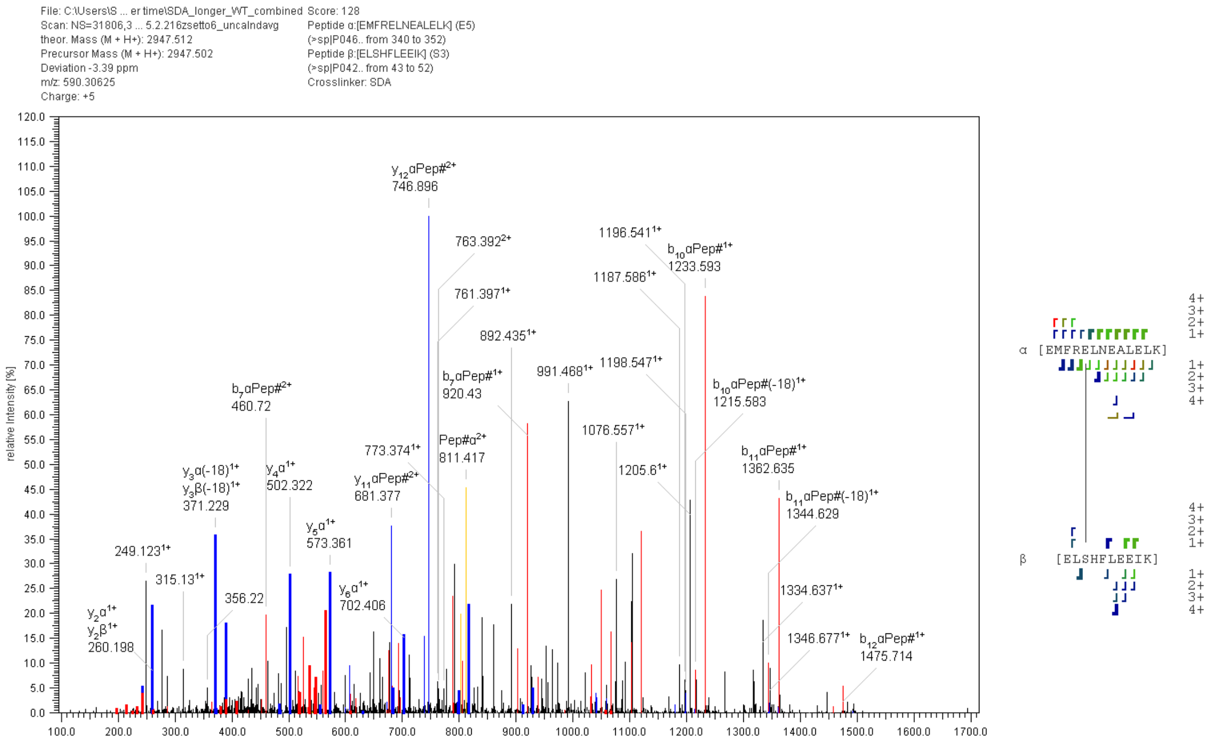

Figure S16. Selected MS/MS spectra and cross-linking assignment of Sulfo-SDA with MeroX.

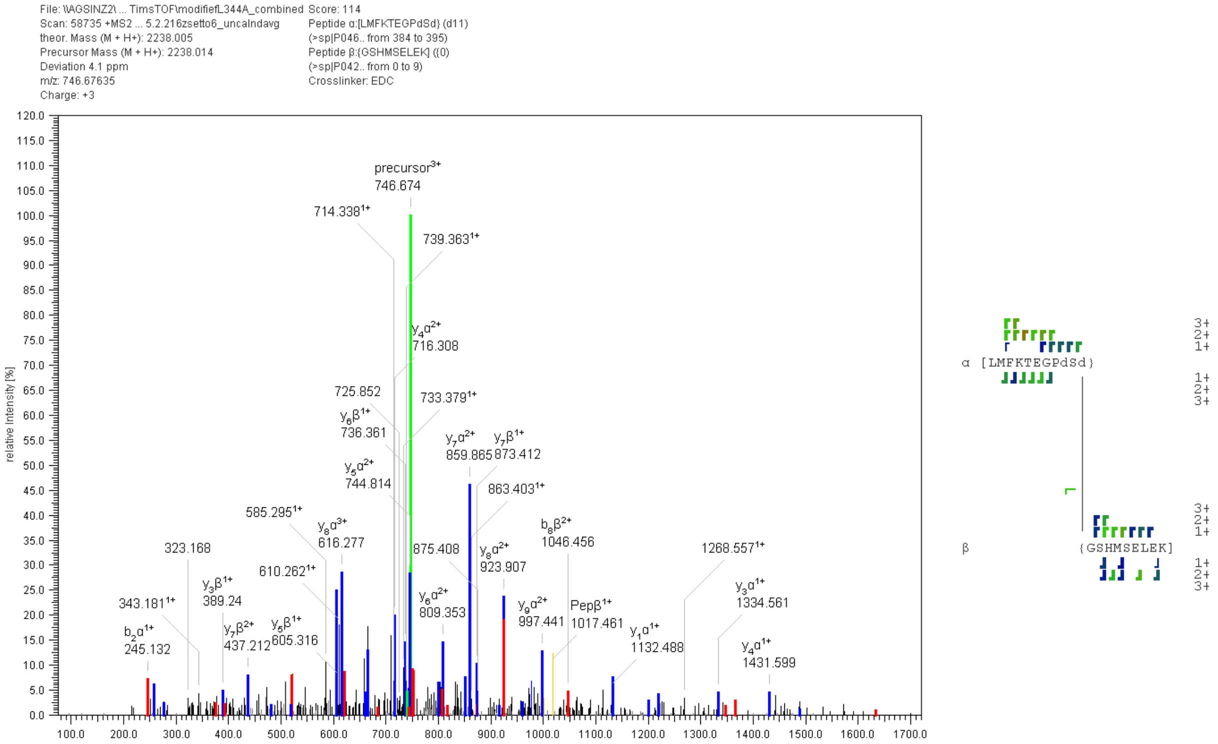

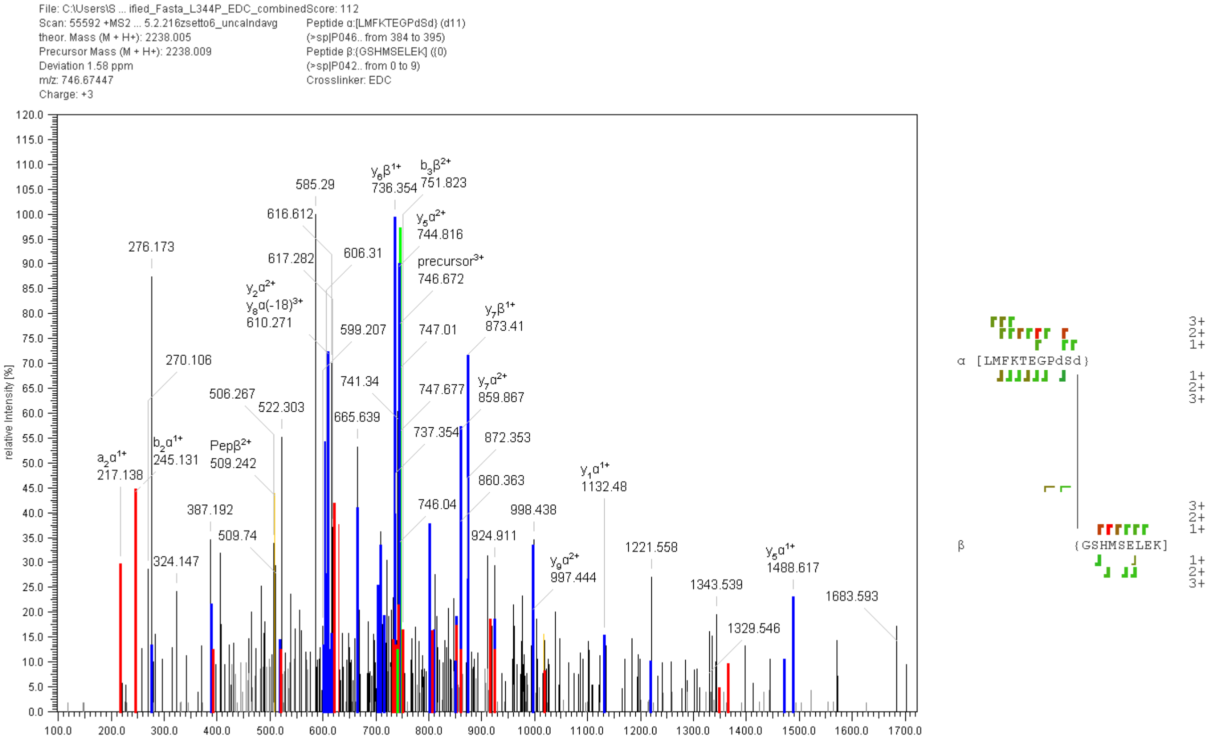

Figure S17. Selected MS/MS spectra and cross-linking assignment of EDC/NHS with MeroX.

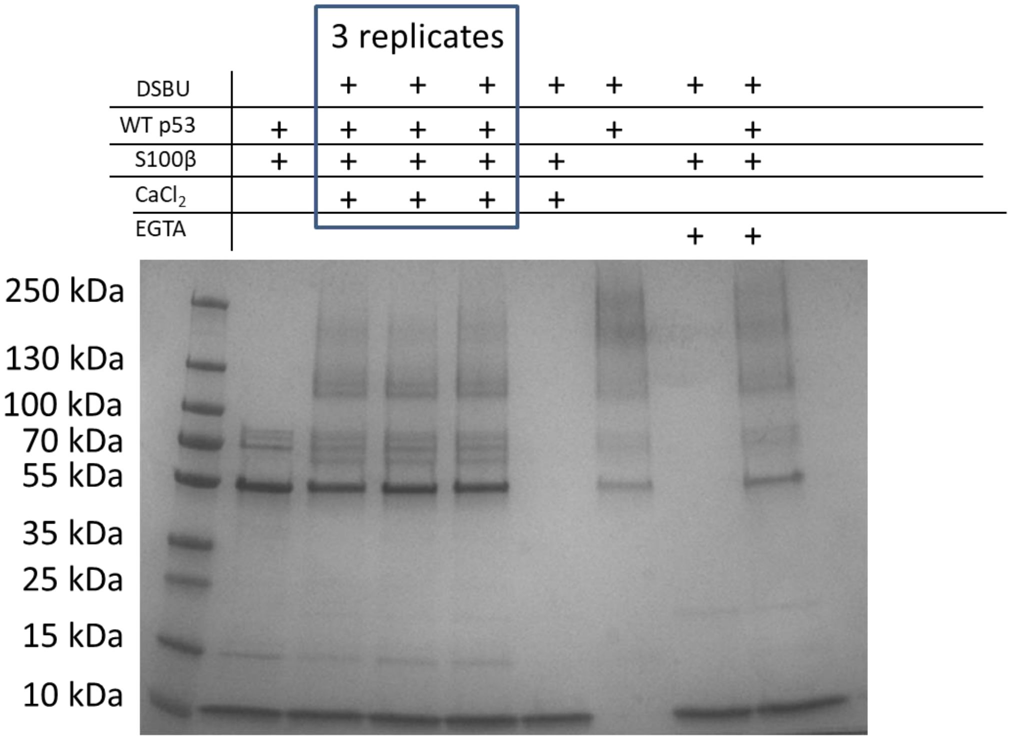

Figure S18. SDS-PAGE (4-20% gradient gel) for cross-linking experiments of recombinant, tag-free S100β and tetrameric wild-type p53 with DSBU.

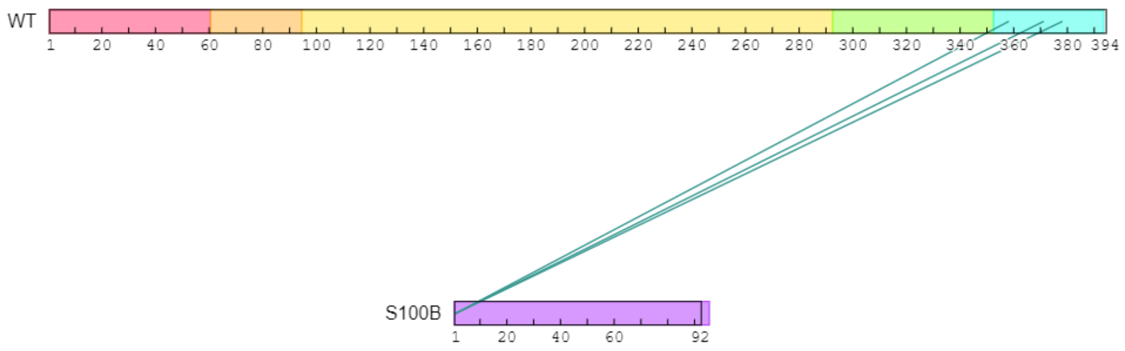

Figure S19. Mapping of DSBU cross-links of recombinant, tag-free S100β and tetrameric wild-type p53. Cross-links are visualized with xiNET[4].

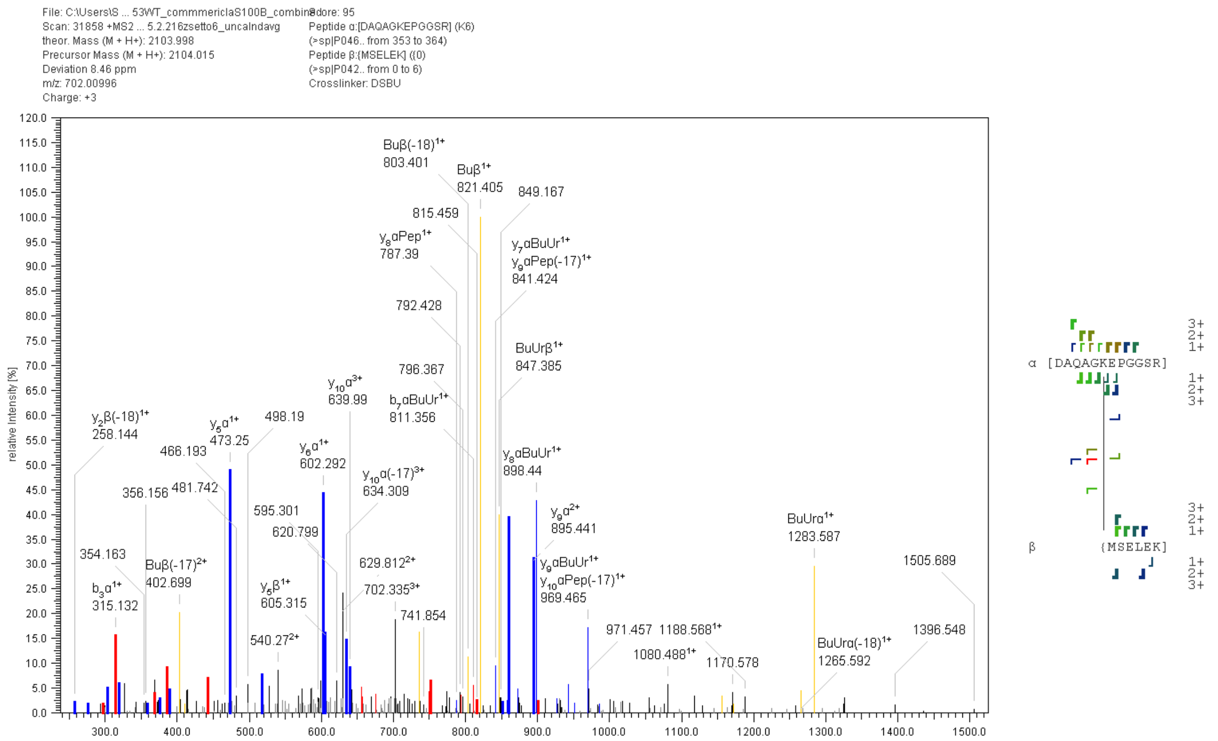

Figure S20. Selected MS/MS spectra and cross-linking assignment of recombinant, tag-free S100β and tetrameric wild-type p53 with DSBU with MeroX.

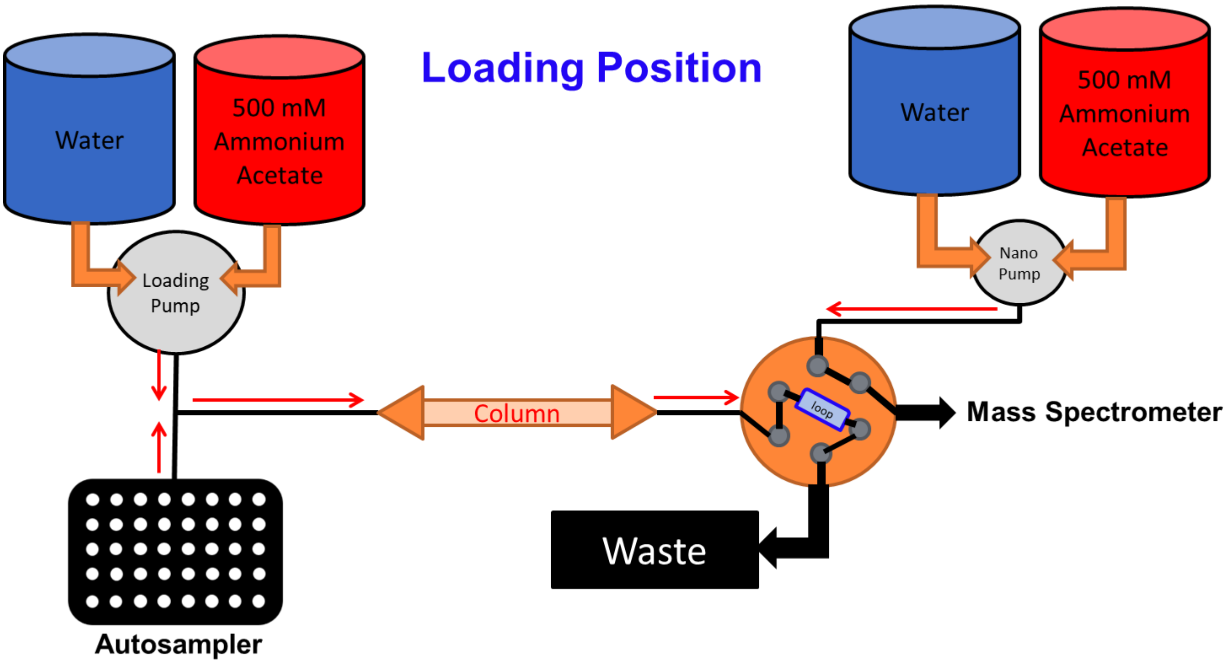

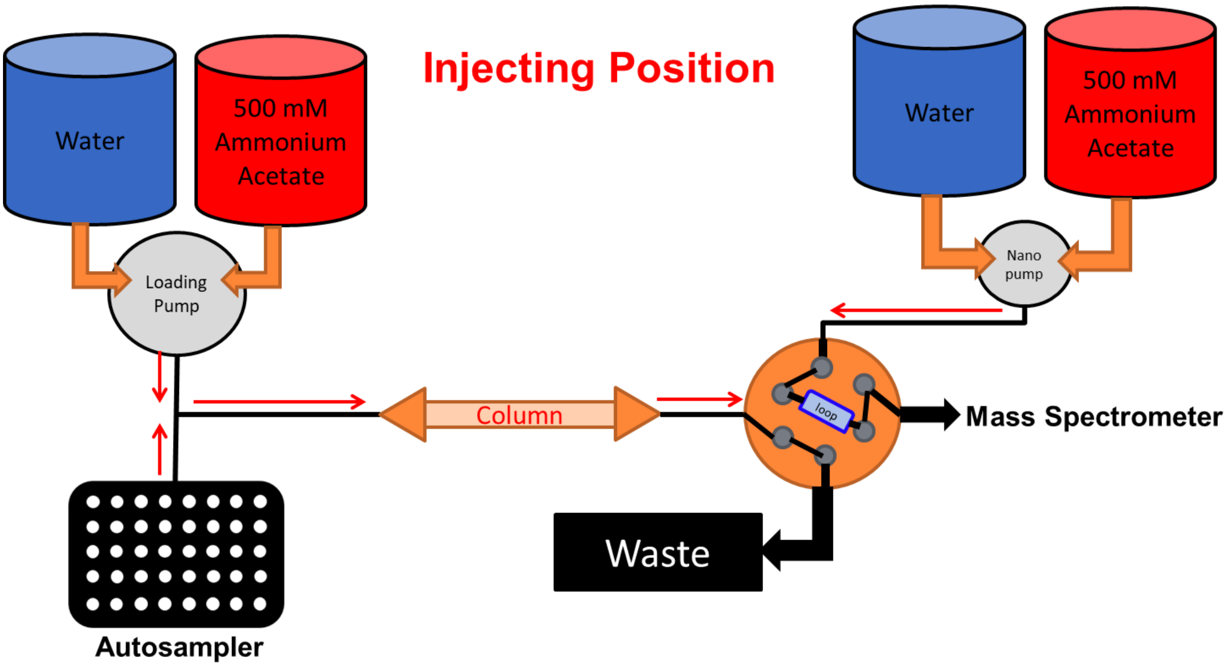

Figure S21. Instrumental setup of the online buffer exchange (OBE) at load and inject position[5].

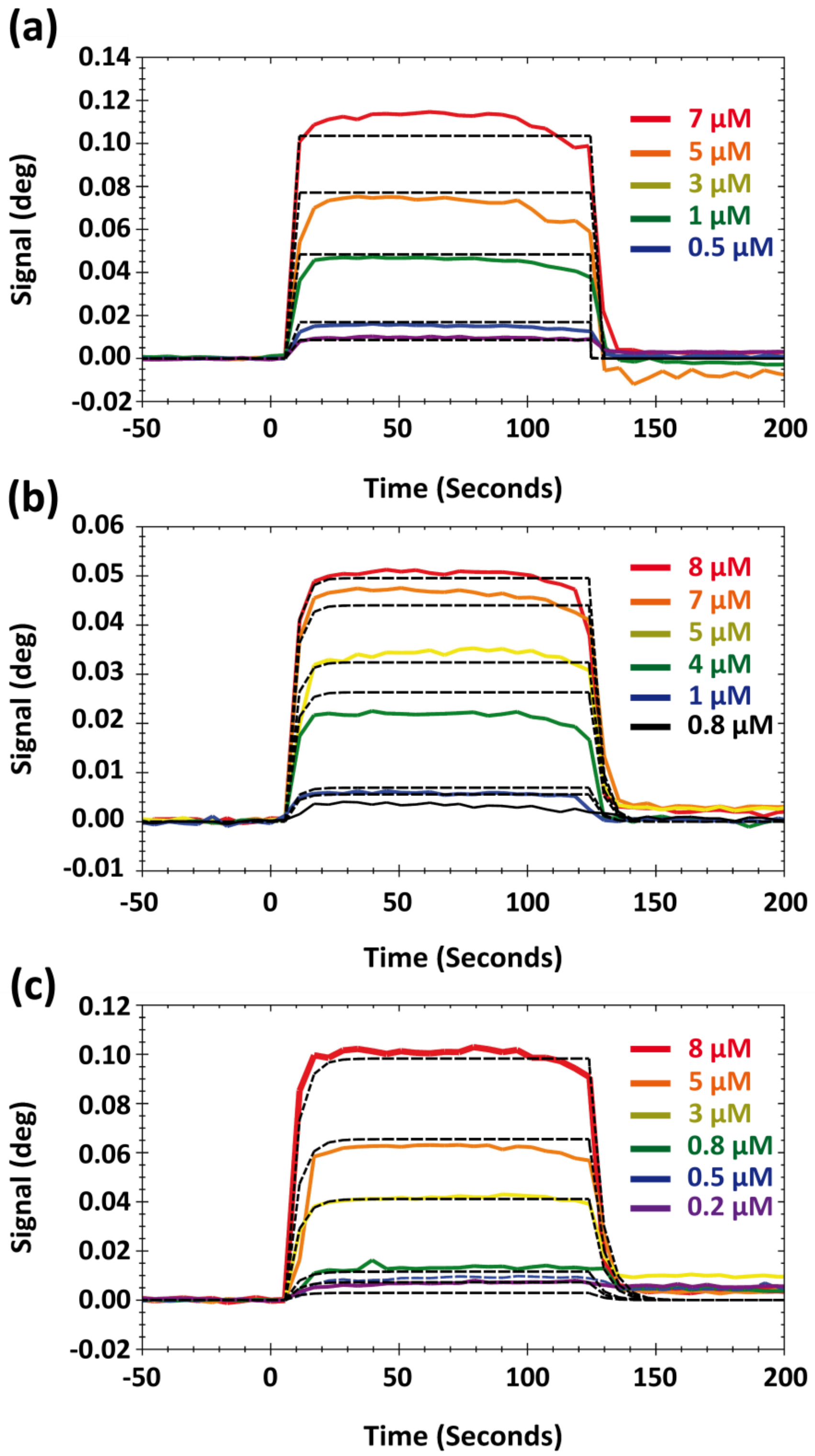

Figure S22. Surface plasmon resonance (SPR) experiments of p53 variants binding to immobilized S100β. (a) Wild-type, tetrameric; (b) L344A dimeric; (c) L344P monomeric p53. Dashed lines represent curve fittings of the one-to-one model. The K_D value of wild-type p53 and S100β is 41.2 ± 0.01 μM. For dimeric p53 (L344A variant) the K_D value is 60.6 ± 0.3 μM and for the monomeric p53 (L344P variant) the K_D value is is 40.3 ± 0.6 μM.

Table S1. Binding affinities (K_D) of reported p53 peptides/S100β and other protein binding partners.

**Interaction**	**Binding affinity (K_D) (μM)**	**Method**
p53 (aa. 293-393)/S100β	0.25 ± 0.05[6]	Fluorescence Anisotropy
p53 (aa. 325-339)/S100β	172 ± 4	
p53 (aa. 325-355)/S100β	112 ± 7	
p53 (aa. 340-351)/S100β	302 ± 7	
p53 (aa. 367-393)/S100β	102 ± 3	
p53 (aa. 305-322)/S100β	570 ± 50	Analytical Ultracentrifugation
p53 (aa. 325-339)/S100β	180 ± 20	
p53 (aa. 325-355)/S100β	77 ± 11	
p53 (aa. 340-351)/S100β	260 ± 20	
p53 (aa. 367-393)/S100β	82 ± 6	
p53 (aa. 379-393)/14-3-3σ	25.7[7]	Isothermal Calorimetry
p53 (aa. 379-393)/14-3-3σ	34.4	Surface Plasmon Resonance
p53-K382acetylated (aa. 372-389)/Sir2Tm	4.3 ± 0.5[8]	Isothermal Calorimetry

Table S2. Theoretical and experimental masses of p53 and complexes where differences are contributed by different numbers of DSBU modifications.

Species	Theoretical Mass (kDa)	Experimental Mass (kDa)
S100β monomer	10.994	11.062
S100β dimer	21.988	22.010
Tetrameric Wild-Type p53/S100β complex	196.948	199.967
Tetrameric Wild-Type p53	174.960	177.432
Dimeric L344A p53/S100β complex	109.468	111.100
Dimeric L344A p53 variant	87.480	88.215
Monomeric L344P p53/S100β complex	65.728	66.430
Monomeric L344P p53 variant	43.740	44.063

Table S3. Summary of cross-links identified between p53 and S100β with cross-linker DSBU.

Wild-Type tetrameric

Peptide 1 (p53)	From	To	Peptide 2 (S100β)	From	To	Site1	Site2
KGQSTSR	374	380	{GSHMSELEK	0	9	K374	*N*-terminus

L344A dimeric

Peptide 1 (p53)	From	To	Peptide 2 (S100β)	From	To	Site1	Site2
KGQSTSR	374	380	{GSHMSELEK	0	9	K374	*N*-terminus
DAQAGKEPGGSR	353	364	{GSHMSELEK	0	9	K358	S5
SKKGQSTSR	372	380	HKLK	29	32	S379	K30

L344P monomeric

Peptide 1 (p53)	From	To	Peptide 2 (S100β)	From	To	Site1	Site2
DAQAGKEPGGSR	353	364	{GSHMSELEK	0	9	K358	*N*-terminus
KGQSTSR	374	380	{GSHMSELEK	0	9	K374	*N*-terminus

Table S4. Summary of the cross-links identified of p53 and S100β with cross-linker sulfo-SDA.

**Wild-Type**							
**Peptide 1 (p53)**	**From**	**To**	**Peptide 2 (S100β)**	**From**	**To**	**Site1**	**Site2**
EMFRELNEALEIK	340	352	ELSHFLEEIK	43	52	E344	S45

Table S5. Summary of the cross-links identified of p53 and S100β with cross-linker EDC/sulfo-NHS.

**Wild-Type tetrameric**							
Peptide 1 (p53)	From	To	Peptide 2 (S100β)	From	To	Site1	Site2
DIEQWFTE	50	57	{GSHMSELEK	0	9	*N*-terminus	D50
DPGPDEAPR	58	66	{GSHMSELEK	0	9	*N*-terminus	D62
TEGPDSD	388	394	{GSHMSELEK	0	9	*N*-terminus	D392
TEGPDSD	388	394	{GSHMSELEK	0	9	*N*-terminus	D394
**L344A dimeric**							
Peptide 1 (p53)	From	To	Peptide 2 (S100β)	From	To	Site1	Site2
DIEQWFTE	50	57	{GSHMSELEK	0	9	*N*-terminus	D50
DPGPDEAPR	58	66	{GSHMSELEK	0	9	*N*-terminus	D62
TEGPDSD	388	394	{GSHMSELEK	0	9	*N*-terminus	D392
TEGPDSD	388	394	{GSHMSELEK	0	9	*N*-terminus	D394
**L344P monomeric**							
Peptide 1 (p53)	From	To	Peptide 2 (S100β)	From	To	Site1	Site2
DDLMLSPD	42	49	{GSHMSELEK	0	9	*N*-terminus	D49
DDIEQWFTE	49	57	{GSHMS	0	5	*N*-terminus	D49
DIEQWFTE	50	57	{GSHMSELEK	0	9	*N*-terminus	D50
DPGPDEAPR	58	66	{GSHMSELEK	0	9	*N*-terminus	D62
LMFKTEGPDSD	384	394	{GSHMSELEK	0	9	*N*-terminus	D392
TEGPDSD	388	394	{GSHMSELEK	0	9	*N*-terminus	D394

Table S6. Summary of the cross-links identified of p53 and recombinant, tag-free S100β with cross-linker DSBU.

**Wild-Type**							
**Peptide 1 (p53)**	**From**	**To**	**Peptide 2 (S100β)**	**From**	**To**	**Site1**	**Site2**
DAQAGKEPGGSR	353	364	{MSELEK	0	6	K358	*N*-terminus
AHSSHLKSK	365	373	{MSELEK	0	6	K371	*N*-terminus
KGQSTSR	374	380	{MSELEK	0	6	T378	*N*-terminus

Literature References

[1] C. Arlt, V. Flegler, C. H. Ihling, M. Schäfer, I. Thondorf, A. Sinz, *Angew. Chem., Int. Ed. Engl.* **2017**, *56*, 275–279.

[2] M. H. Clare, Rekombinante Expression von S100ß Und Interaktionsstudien Mit Dem Tumorsupressor P53, Martin-Luther Universität Halle-Wittenberg (Germany), **2018**.

[3] C. Iacobucci, C. Piotrowski, R. Aebersold, B. C. Amaral, P. Andrews, K. Bernfur, C. Borchers, N. I. Brodie, J. E. Bruce, Y. Cao, S. Chaignepain, J. D. Chavez, S. Claverol, J. Cox, T. Davis, G. Degliesposti, M. Q. Dong, N. Edinger, C. Emanuelsson, M. Gay, M. Götze, F. Gomes-Neto, F. C. Gozzo, C. Gutierrez, C. Haupt, A. J. R. Heck, F. Herzog, L. Huang, M. R. Hoopmann, N. Kalisman, O. Klykov, Z. Kukačka, F. Liu, M. J. Maccoss, K. Mechtler, R. Mesika, R. L. Moritz, N. Nagaraj, V. Nesati, A. G. C. Neves-Ferreira, R. Ninnis, P. Novák, F. J. O'Reilly, M. Pelzing, E. Petrotchenko, L. Piersimoni, M. Plasencia, T. Pukala, K. D. Rand, J. Rappsilber, D. Reichmann, C. Sailer, C. P. Sarnowski, R. A. Scheltema, C. Schmidt, D. C. Schriemer, Y. Shi, J. M. Skehel, M. Slavin, F. Sobott, V. Solis-Mezarino, H. Stephanowitz, F. Stengel, C. E. Stieger, E. Trabjerg, M. Trnka, M. Vilaseca, R. Viner, Y. Xiang, S. Yilmaz, A. Zelter, D. Ziemianowicz, A. Leitner, A. Sinz, *Anal. Chem.* **2019**, *91*, 6953–6961.

[4] C. W. Combe, L. Fischer, J. Rappsilber, *Mol. Cell. Proteomics* **2015**, *14*, 1137–1147.

[5] Z. L. VanAernum, F. Busch, B. J. Jones, M. Jia, Z. Chen, S. E. Boyken, A. Sahasrabuddhe, D. Baker, V. H. Wysocki, *Nat. Protoc.* **2020**, *15*, 1132–1157.

[6] M. R. Fernandez-Fernandez, D. B. Veprintsev, A. R. Fersht, *Proc. Natl. Acad. Sci. U. S. A.* **2005**, *102*, 4735–4740.

[7] A. Kuusk, J. F. Neves, K. Bravo-Rodriguez, A. Gunnarsson, Y. B. Ruiz-Blanco, M. Ehrmann, H. Chen, I. Landrieu, E. Sanchez-Garcia, H. Boyd, C. Ottmann, R. G. Doveston, *ACS Chem. Biol.* **2020**, *15*, 262–271.

[8] M. S. Cosgrove, K. Bever, J. L. Avalos, S. Muhammad, X. Zhang, C. Wolberger, *Biochemistry* **2006**, *45*, 7511–7521.
